# Functional characterization of the *Hyles euphorbiae* hawkmoth transcriptome reveals strong expression of phorbol ester detoxification and seasonal cold hardiness genes

**DOI:** 10.1186/s12983-018-0252-2

**Published:** 2018-05-01

**Authors:** M. Benjamin Barth, Katja Buchwalder, Akito Y. Kawahara, Xin Zhou, Shanlin Liu, Nicolas Krezdorn, Björn Rotter, Ralf Horres, Anna K. Hundsdoerfer

**Affiliations:** 1 Museum of Zoology, Senckenberg Natural History Collections Dresden, Koenigsbruecker Landstrasse 159, D-01109 Dresden, Germany; 2 Florida Museum of Natural History, University of Florida, Gainesville, FL 32611 USA; 3 Department of Entomology, China Agricultural University, Bejing, 100193 China; 4 China National Gene Bank, Beijing Genomics Institute, Shenzhen, 518083 China; 5 GenXPro GmbH, Altenhöferallee 3, D-60438 Frankfurt am Main, Germany

**Keywords:** Diapause, Diterpene ester, Freeze-avoidance, Functional ecology, Functional traits, Lepidoptera, RNA-Seq, Sphingidae, TPA, Winter biology

## Abstract

**Background:**

The European spurge hawkmoth, *Hyles euphorbiae* (Lepidoptera, Sphingidae), has been intensively studied as a model organism for insect chemical ecology, cold hardiness and evolution of species delineation. To understand species isolation mechanisms at a molecular level, this study aims at determining genetic factors underlying two adaptive ecological trait candidates, phorbol ester (TPA) detoxification and seasonal cold acclimation.

**Method:**

A draft transcriptome of *H. euphorbiae* was generated using Illumina sequencing, providing the first genomic resource for the hawkmoth subfamily Macroglossinae. RNA expression levels in tissues of experimental TPA feeding larvae and cooled pupae was compared to levels in control larvae and pupae using 26 bp RNA sequence tag libraries (DeepSuperSAGE). Differential gene expression was assessed by homology searches of the tags in the transcriptome.

**Results:**

In total, 389 and 605 differentially expressed transcripts for detoxification and cold hardiness, respectively, could be identified and annotated with proteins. The majority (22 of 28) of differentially expressed detox transcripts of the four ‘drug metabolism’ enzyme groups (cytochrome P450 (CYP), carboxylesterases (CES), glutathione S-transferases (GST) and lipases) are up-regulated. Triacylglycerol lipase was significantly over proportionally annotated among up-regulated detox transcripts. We record several up-regulated lipases, GSTe2, two CESs, CYP9A21, CYP6BD6 and CYP9A17 as candidate genes for further *H. euphorbiae* TPA detoxification analyses. Differential gene expression of the cold acclimation treatment is marked by metabolic depression with enriched Gene Ontology terms among down-regulated transcripts almost exclusively comprising metabolism, aerobic respiration and dissimilative functions. Down-regulated transcripts include energy expensive respiratory proteins like NADH dehydrogenase, cytochrome oxidase and ATP synthase. Gene expression patterns show shifts in carbohydrate metabolism towards cryoprotectant production. The Glycolysis enzymes, G1Pase, A1e, Gpi and an Akr isoform are up-regulated. Glycerol, an osmolyte which lowers the body liquid supercooling point, appears to be the predominant polyol cryoprotectant in *H. euphorbiae* diapause pupae. Several protein candidates involved in glucose, glycerol, myo-inositol and potentially sorbitol and trehalose synthesis were identified.

**Conclusions:**

A majority of differently expressed transcripts unique for either detoxification or cold hardiness indicates highly specialized functional adaptation which may have evolved from general cell metabolism and stress response.The transcriptome and extracted candidate biomarkers provide a basis for further gene expression studies of physiological processes and adaptive traits in *H. euphorbiae*.

**Electronic supplementary material:**

The online version of this article (10.1186/s12983-018-0252-2) contains supplementary material, which is available to authorized users.

## Background

*Hyles euphorbiae* (Linnaeus 1758) (Lepidoptera, Sphingidae) is intensively studied as a model species for insect chemical ecology [1], seasonal cold hardiness [2–5] and species delineation, including hybridization and population structure [6–9]. However, the lack of genomic or transcriptomic data has hampered studies of underlying molecular mechanisms. The Western Palearctic *Hyles euphorbiae* complex (HEC [8]) includes five valid species traditionally defined by geographic occurrence [10]. However, geographic occurrence is neither discrete nor stable in time, denoting a scenario of ongoing hybridization and/or incomplete lineage sorting [8, 9]. A recent study reveals that the HEC may rather be a single, genetically distinct biological species that has two morphologically and ecologically distinct subgroups [11], while discrepancies between taxon distribution ranges, morphotypes [7, 12] and mitochondrial lineages [8] remain. The HEC primary Central European species, *H. euphorbiae*, is characterized by two particularly interesting ecological adaptations: The detoxification of diterpen-ester containing food plants and the survival of harsh winters. This makes it distinct from other HEC species/lineages and an ideal case to investigate functional isolation mechanisms in speciation processes in the HEC and in general.

Larvae of numerous *Hyles* species specialize to feed on different food plant species of *Euphorbia* Linnaeus 1753 (Euphorbiaceae) [13]. Plants in this genus contain diterpene esters, mainly phorbol (ingenol) esters – toxic skin irritants [14] with antifeedant properties [15]. Polymorphic HEC larvae advertise their toxicity by exhibiting aposematic coloration, presumably based on utilizing *Euphorbiae* chemicals for defense [1]. *Euphorbia* species differ strongly in their spectra of diterpene esters [16], which may account for differential detoxification abilities of *Hyles* species specialized on different *Euphorbia* species (Hundsdoerfer et al., unpublished, submitted to Journal of Chemical Ecology). The most common larval food plant of *H. euphorbiae* in Central Europe is *E. cyparissias*, which contains a structural isomer of the particularly active and irritant phorbol ester, 12-tetradecanoyl-phorbol-13-acetate (TPA), while no TPA occurs in *E. myrsinites*, *E. paralias* and *E. segetalis* [16], the three main food plants of the HEC in southern Europe. The phorbol ester binding site of the target protein kinase C [17] shows the same conserved nucleotide sequence in both *H. euphorbiae* and *Hippotion celerio* (Hundsdoerfer et al., unpublished, submitted to Journal of Chemical Ecology), the latter being a phorbol ester sensitive relative of *Hyles* [6]. We thus assume that intoxication is prevented by an active detoxification system. Larval feeding experiments have shown that *H. euphorbiae* larvae are insensitive to TPA intoxication by oral ingestion and metabolized TPA [1] in captivity, while the responsible mechanism is unknown. In contrast, non-*Euphorbia* feeding larvae of several other *Hyles* species close to the HEC are intoxicated by TPA ingestion (e.g., *H. centralasiae*, *H. vespertilio*; Hundsdoerfer et al., unpublished, submitted to Journal of Chemical Ecology). Thus, the ability to detoxify TPA may represent a key adaptation in speciation processes within the genus *Hyles*. Before being able to test this idea, it is necessary to understand the molecular determinants of TPA detoxification in *H. euphorbiae*. Resistance of insects to naturally occurring plant allelochemicals typically involves increases in the metabolic capabilities of detoxificative enzymes [18]. Esterases, cytochrome P450 monooxygenases (P450s), and glutathione-S transferases (GSTs) have been identified as important resistance mechanisms. We hypothesize some of these also to apply to *H. euphorbiae* and thus focused on these enzyme classes.

Another functional trait that most likely determines the distribution range of *H. euphorbiae* from Central Europe to Central Asia, and its spatial delimitation from other HEC species, is the ability to survive cold winters [2, 3, 8, 12]. Seasonal cold hardiness in *H. euphorbiae* is achieved by pupal developmental diapause initiated in response to short autumn photoperiods [19, 20], in combination with cold acclimation of the diapause pupae over several weeks with lowering temperatures [2]. In contrast, the widely distributed Southern European and Northern African HEC species, *H. tithymali*, may develop diapause pupae during dry season, but those are not cold hardy, and subzero temperatures greatly decreases pupal survival probability [8, 12, 21]. For example, Mende & Hundsdoerfer [9] showed a fluctuating distribution of the northern most *H. tithymali* populations correlating with minimal winter temperature.

Seasonal cold acclimation in insects is often correlated with long-term physiological changes [22, 23] resulting in the accumulation of cryoprotective substances that lower the supercooling point (SCP; i.e., the freezing temperature of body liquid [24], and freeze avoidance or tolerance strategies [22]). Unfortunately, the exact mechanism and strategy in *H. euphorbiae* is unknown, but early work aiming at introducing the species as biological control agent of *E. cyparissias* in Canada indicate a lowered SCP in diapause pupae, but 50% mortality below − 10 °C [3]. Thus, acclimation and freezing is relevant for survival, and we assume a freeze avoidance strategy. More early work indicates physiological changes in metabolic rate [25], carbohydrate metabolism [4, 5] and the accumulation of uric acid [26] and free amino acids [27] in cold acclimated *H. euphorbiae* diapause pupae. These results are congruent with common physiological changes and cryoprotectant production observed in insect seasonal cold hardiness studies [22, 24, 28, 29].

More recently, Stuckas et al. [2] used proteomics and RNA sequence tags to examine the functional molecular mechanisms that underlie *H. euphorbiae* cold acclimation, and identified candidate biomarkers related to oxidative stress response, metabolic changes, translation and biosynthesis. However, due to the lack of genomic and transcriptomic data, their results provided limited potential of gene cloning and amplifying DNA to link potential molecular and physiological adaptations. The closest hawkmoth relative of *Hyles* with extensive genomic data available is *Manduca sexta* (Manduca Base, www.insect-genome.com/data/detail.php?id=14), although curated protein annotations are limited [30].

Here we used RNA-Seq, de novo assembly and functional annotation in collaboration with the 1000 Insect Transcriptome Evolution (1KITE) consortium (www.1KITE.org) [31] to provide the first draft transcriptome for *H. euphorbiae* and the hawkmoth subfamily Macroglossinae. Using these data as references, we analyzed differential gene expression using DeepSuperSAGE sequencing of RNA-tag libraries [2, 32] focusing on TPA detoxification and seasonal cold hardiness in *H. euphorbiae*. If adaptive molecular mechanisms of the two ecological traits are linked to gene expression profiles, we expect to find trait-specific transcript regulation. The aim is to postulate regulated transcripts as potential candidates underlying functional differences with respect to species delimitation within the HEC.

## Results and discussion

### Transcriptome assembly

**Fig. 1:**
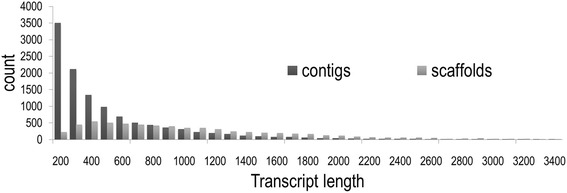
Length distribution of contigs and scaffolds among all assembled *H. euphorbiae* transcripts

In total, 21,656,040 raw reads were generated (1-KITE library ID: INSinlTAYRAAPEI-56). After quality trimming, 17,257,056 clean 150 bp reads were assembled into a final *H. euphorbiae* transcriptome of 18,080 transcripts, consisting of 11,514 singleton contigs and 6566 scaffolds (Table 1). Of the scaffolds, 3443 clustered into 1445 isogroups (mean = 2.38 scaffolds per isogroup), so that the total assembly contained 16,082 unigenes (Table 1). Mean transcript length was 776 bp (992 bp if considering only annotated transcripts), ranging between 200 and 5412 bp. Roughly 75% of all transcripts ranged between 200 and 1000 bp, with scaffolds making up a large part of the long transcripts (Fig. 1). An N50 value of 1084 bp (1338 bp for annotated transcripts), above average transcript length, was indicative of a fair amount of large informative transcripts, and an efficient assembly of the transcriptome data.

**Table 1:** Summary statistics of the Illumina HiSeq 2000 *H. euphorbiae* transcriptome assembly and annotation

Raw no. of sequenced reads	21,656,040
No. of reads after quality control	17,257,056
Total assembly length [bp]	14,027,954
Q20 [%]^a^	89.52
Proportion of Ns [%]	0.02
GC content [%]	39.30
Mean abundance [k-mers] (min – max)^b^	16.17 (3 – 165)
No. of transcripts	18,080
No. of singleton contigs	11,514
No. of isogroups	1445
No. of unigenes	16,082
Mean no. transcripts per isogroup (min – max)	2.38 (2 – 5)
Mean length of transcripts [bp] (min – max)	775.89 (200 – 5412)
N50^c^ [bp]	1084
No. of transcripts with BLASTX hits^d^	8289
No. of transcripts with GO annotations^d^	7070
No. of transcripts with EC/KEGG annotations^d^	2806
No. of GO annotated transcripts with tag hits^d, e^	4451

^a^Phred Quality Score

^b^Mean coverage of k-mers that support a k + 1 length (= 32 bp) of a transcript

^c^minimal length of the set of transcripts that makes up half of the total assembly length

^d^all BLASTX hits with *e* < 10^− 5^ and a sequence similarity of > 55%

^e^transcripts that where a best BLASTN hit (bit score > 40) for DeepSuperSAGE tags

The number of 16,082 *H. euphorbiae* unigenes fits those known from several distantly related Lepidoptera genomes, ranging from 12,669 in *Heliconius melpomene* (Nymphalidae) to 18,071 in *Plutella xylostella* (Plutellidae) [33, 34]. The number of predicted protein-coding genes is also in the same range as the closely related *M. sexta*, which is known to have 15,551 (www.insect-genome.com/data/detail.php?id=14). This suggests that our transcriptome probably contains at least partial transcripts of most coding genes, even though our number of unigenes might be partially overestimated, if not all isoforms clustered properly into isogroups. However, the *M. sexta* gene set contains 27,402 protein sequences if alternative splicing and isoforms are included. Thus, some alternative protein forms are perhaps missing in the *H. euphorbiae* transcriptome, probably because we only included life stages and environmental conditions relevant for this study. Moreover, within the 1KITE framework, sequencing was not tissue-specific and relatively shallow, with roughly 20 million reads and a mean abundance (k-mer coverage) of 16.17 (Table 1), so that tissue-specific expressed transcripts may have been missed.

### Functional annotation

Of the 18,080 *H. euphorbiae* transcripts, 8289 (46%) could be identified by BLASTX hits in the non-redundant (nr) NCBI protein database with *e* < 10^− 5^ and a similarity > 55% (Additional file 1: Figure S1, Additional file 2: Table S1). This relatively low number of significant protein annotations*,* despite a fair amount of curated genomic Lepidoptera data available (e.g., ButterflyBase; www.seriport.in/butterflybase/), suggests a high genomic divergence of *H. euphorbiae* from the existing well-studied model species. The nr database contains the RefSeq dataset encompassing all available reference genomes, therefore phylogenetically distant genomes were included in the annotation procedure. Unfortunately, most non-model Lepidoptera transcriptomes are not protein-annotated [33, 35], and annotation success is typically ≤50% for these species and other insect de novo transcriptomes [36–40] due to the lack of closely related reference genomes. Thus, characterizing proteins from more diverse species is important for future studies.

**Fig. 2:**
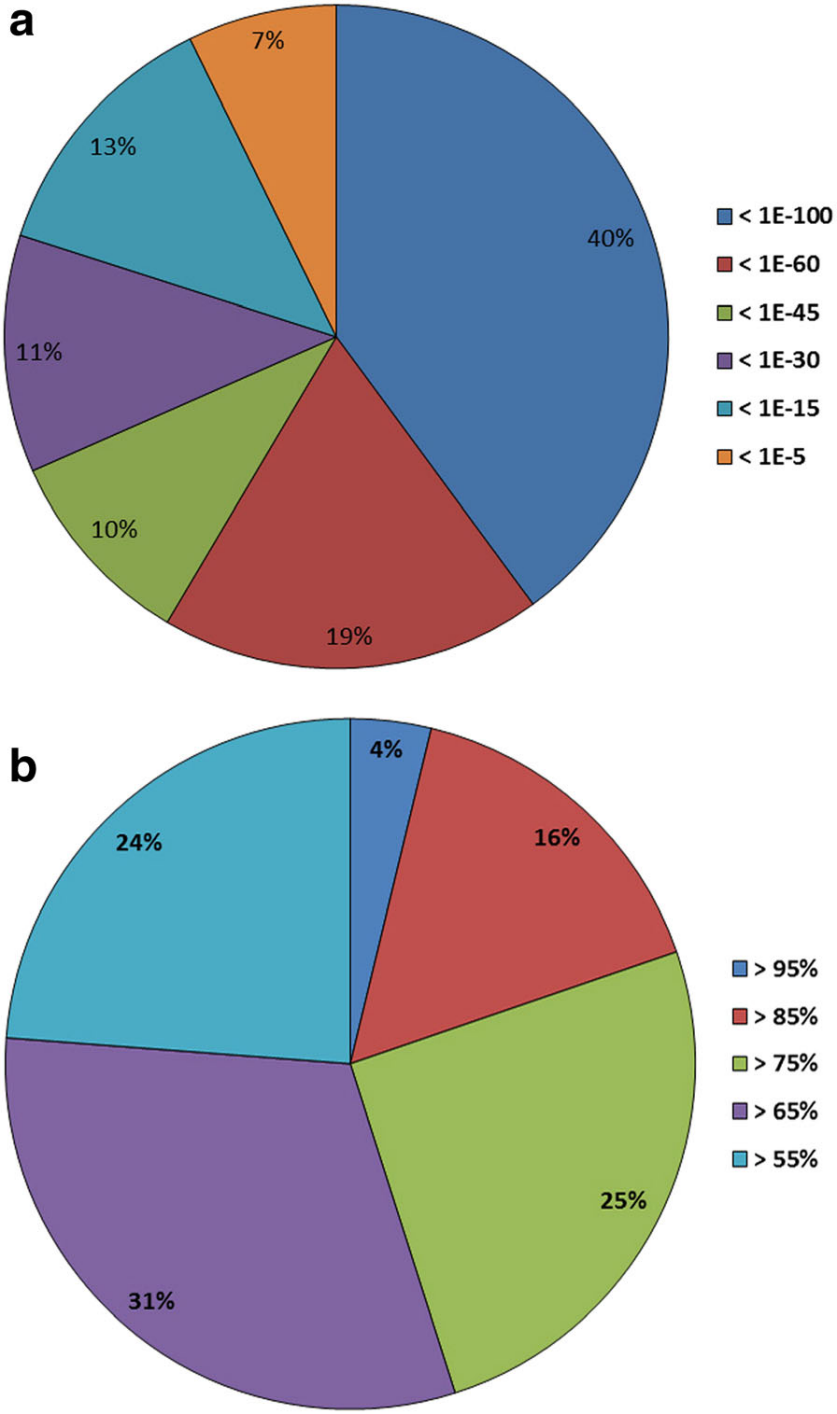
Quality statistics of best BLASTX hits. All *H. euphorbiae* transcripts with *e* < 10^− 5^ and a similarity > 55% are covered. **a** *e*-value **b** sequence similarity distribution

The *e*-value frequency distribution among the annotated transcripts revealed that the majority (59%) of the best BLASTX hits had a very high alignment quality with *e* < 10^− 60^ (Fig. 2a), but only 20% had sequence similarities > 85% with the respective transcript (Fig. 2b). This confirms a rather distant relationship of *H. euphorbiae* with most of its best matches in the NCBI nr protein database. Reducing the similarity threshold to 40% increases the annotation success to 52% (9464 transcripts), but may increase the number of false positives. Most nr annotations (95%) are from Lepidoptera sequences (Additional file 3: Figure S2), with 5165 transcripts having strong homology with the Silkworm *Bombyx mori* (Saturnidae; 60% of all and 63% of Lepidoptera annotated transcripts; Additional file 3: Figure S2), which is the best studied Lepidoptera model for genomic data (SilkDB v2.0; http://silkworm.genomics.org.cn/). Only about 5% of all annotations were BLASTX hits with *M. sexta*, a closer relative of *H. euphorbiae* (both belong to the family Sphingidae). In contrast, running the BLASTX search only against Lepidoptera orthologs (see below) resulted in 75% *M. sexta* hits, although most of them were non-annotated predicted proteins. Thus, current curative progress in Manduca Base will allow for improved transcriptome annotation of *Hyles* and other non-model Lepidoptera.

For additional functional annotation, Gene Ontology (GO) terms were mapped to the 8289 annotated *H. euphorbiae* transcripts (Table 1, Additional file 2: Table S1). ANNEX augmentation improved the initial mapping only by less than 1% to a total of 40,619 GO terms (5887 unique GO terms) assigned to 7070 (~ 39%) transcripts (Table 1, Additional file 2: Table S1). Furthermore, functional annotation resulted in the assignment of 2806 transcripts (16%) with 513 different enzymes, based on enzyme commission numbers (EC), contained in 125 different pathways of the Kyoto Encyclopedia of Genes and Genomes (KEGG) (Table 1, Additional file 4: Table S2). However, 69% (5759) of the annotated proteins were functionally ambiguous, containing words such as “hypothetical”, “predicted”, “putative”, “uncharacterized”, “unknown” or “unnamed”, which was also observed in other insect transcriptomes [38, 39]. Filtering out these so called “bad words” and replacing them with the next best BLASTX hit using a Python™ script [41] resulted in less significant hits with a much broader spectrum of more distantly related species. Thus, we kept the original transcriptome annotations including ambiguities as best possible output for further analyses.

**Fig. 3:**
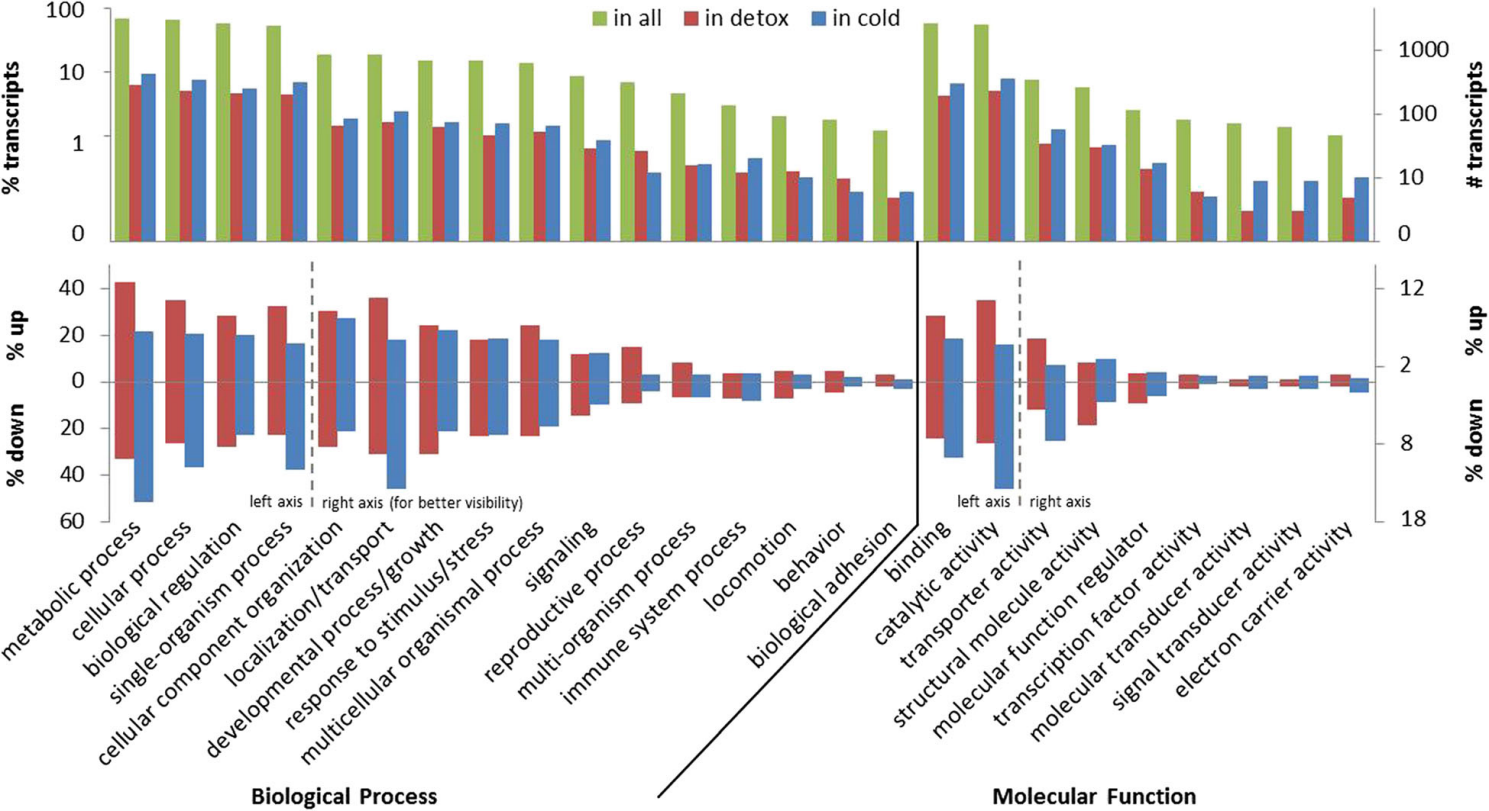
Distribution of Gene Ontology (GO) terms. Second level GO categories of biological process and molecular function among functionally annotated *H. euphorbiae* transcripts with BLASTN hits within DeepSuperSAGE tag libraries (only GO terms annotated to > 1% of all transcripts with UniTag hits). The upper panel shows the proportion and count of GO annotations in all (4451) and in differentially expressed (816) transcripts, while the lower panel shows proportional GO annotation among up- and down-regulated transcripts in libraries of the two experiments (detoxification and cold hardiness)

All GO terms were distributed among the three main GO categories biological process (53%), molecular function (22%) and cellular component (25%). In total 4451 functionally annotated transcripts (~ 25% of all transcripts) were best BLASTN hits to 26 bp mRNA tags from the DeepSuperSAGE libraries and could therefore be used for differential gene expression and enrichment analyses of the TPA and cold acclimation experiments (see below). The 25 s level GO subcategories that were annotated to at least 1% of those transcripts are summarized in Fig. 3. Most frequent GO terms were “cellular process” and “metabolic process” among biological process terms, and “binding” and “catalytic activity” among molecular function. This GO term distribution pattern is similar to that of other Lepidoptera transcriptomes [39, 40, 42], indicating typical general metabolic gene expression. However, while these general metabolic terms tend to be over-represented among the detoxification up-regulated transcripts, they are over-represented among the down-regulated transcripts of the cold treatment (Fig. 3). Such an increase in metabolic activity is expected after initiation of detoxification mechanisms, while metabolic down-regulation is typical for cold hardy organisms in diapause [29, 43].

### Transcriptome completeness

Of all transcripts, 83% (14,958) and 99% of all protein annotated transcripts contained open reading frames, ORFs ≥60 codons (180 – 4722 bp of the longest ORF per transcript; median 366 bp) or interPro domains (Additional file 2: Table S1), confirming coding gene annotations [44]. In addition, 69% of transcripts lacking significant protein annotations had ORFs or interPro domains, suggesting a considerable number of coding genes are still to be identified, which appear to be *Hyles-*specific and not a homolog to the core set of insect gene orthologs identified within the 1KITE project [31]. However, not all ORFs are necessarily translated and untranslated mRNA regions are common in eukaryotes [44]. Moreover, many short ORFs (median: 240 bp) among the unannotated transcripts suggest false positives to some extent, particularly in the relatively AT rich (61%) transcriptome, in which random stop codons are likely (CLC Genomics Workbench User Manual). In contrast, ORFs of the annotated transcripts had lengths (median: 570 bp) well above the overall median.

CEGMA mapped 69% of the 458 core eukaryotic genes (79% partially), which is acceptable considering the mean abundance of 16.17 k-mers (Table 1) among the *H. euphorbiae* transcriptome [45]. The reverse conclusion is that an estimated 21% to 31% of *H. euphorbiae* transcripts are potentially missing or only poorly or partially assembled – we expect these to refer to genes with low expression levels. Transcripts without protein annotations are short (549 bp mean length compared to 776 bp total average), which may have hindered better BLASTX annotation. We predict that future studies including greater sequencing depth and more life stage samples may increase number, coverage and annotation success of unigenes for *H. euphorbiae*.

The Ortholog Hit Ratio (OHR) [42] was calculated from 8195 hits against Lepidoptera-specific BLAST databases (99% of all nr annotated transcripts) (see methods and Additional file 5: Figure S3A). *H. euphorbiae* transcripts had a mean OHR of 0.63 (i.e., on average, 63% of the length of those of major Lepidoptera models), and 11% (868) had OHRs above 1 (i.e., were longer than their database counterpart). The mean OHR was reduced by 16% (1326) due to transcripts having OHRs below 0.2 that probably refer to the partially or weakly assembled coding regions. This is confirmed by a median transcript expression level of 3.26 FPKM (fragments per kilobase of transcript times millions mapped fragments) [46] of those transcripts with low OHRs, compared to a median FPKM of 6.11 of all protein annotated transcripts, after mapping the Illumina reads back to the transcriptome (CLC Genomics Workbench).

Altogether, comparisons of our de novo transcriptome completeness to other transcriptome assemblies of non-model taxa shows a similar or even higher ORF proportion (others have approximately 70% ORF proportion [37, 39]), as well as a similar OHR [39, 42]. The CEGMA score is only slightly below Lepidoptera models with full genomes available [34], suggesting that our *H. euphorbiae* transcriptome is a comparably solid first draft backbone. Deeper sequencing of more tissue and life stage specific biological replicates will complete our data in the near future.

### Differential gene expression and enrichment analyses

**Fig. 4:**
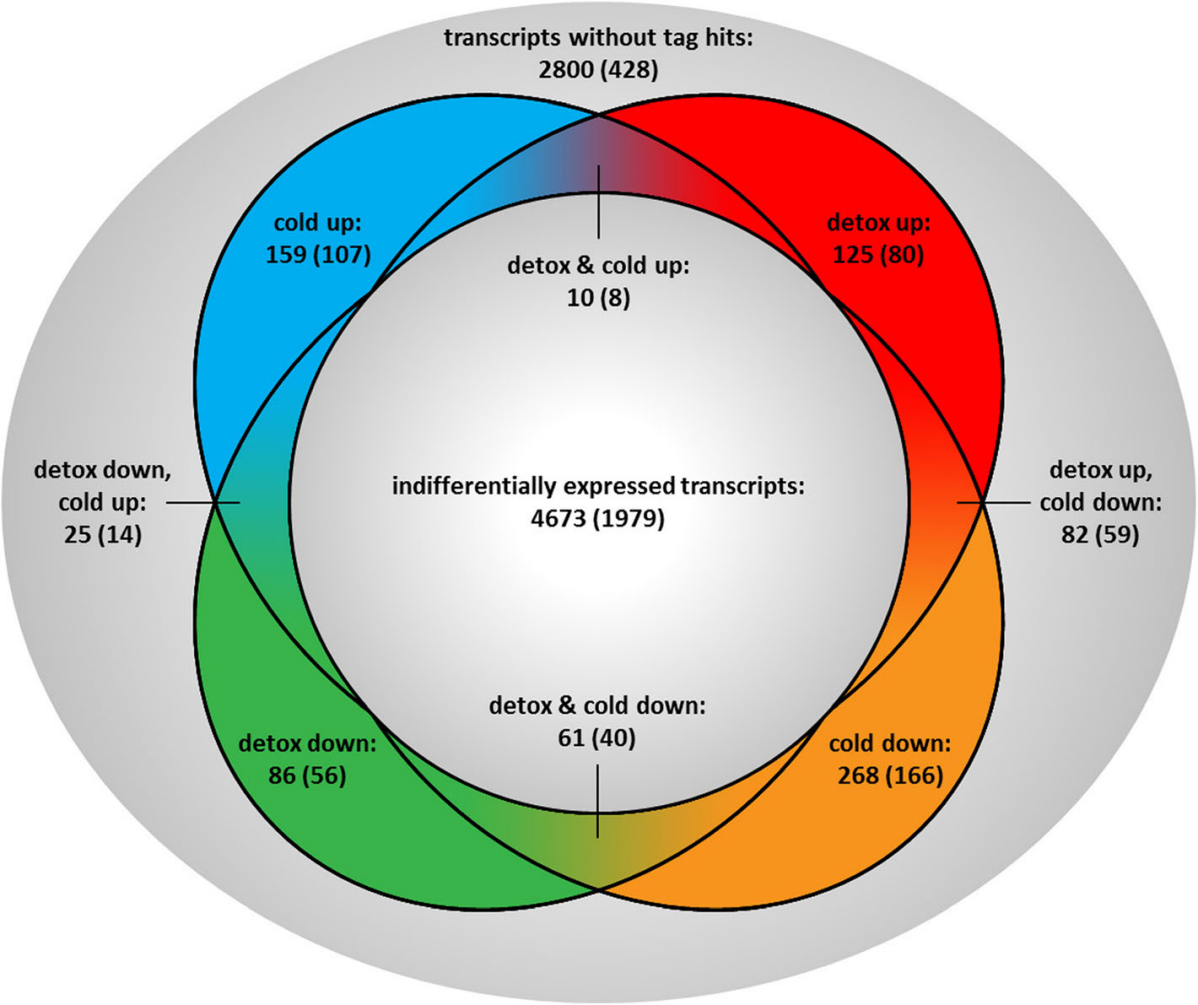
Venn diagram showing differentially expressed transcript distribution. In total, 5489 best hit transcripts (number of unigenes given in brackets) for DeepSuperSAGE tags were assessed, of which 810 GO annotated transcripts with open reading frames showed differential expression in the two treatments (detox = detoxification, cold = cold acclimation)

In order to assess differentially expressed candidate genes in TPA (detox) and cold treatment of *H. euphorbiae* larvae and pupae, we ran a DeepSuperSAGE analysis of 26 bp mRNA tags [2, 32] and a BLASTN search of those tags against the assembled transcriptome (Table 2, Additional file 6: Figure S4). This resulted in 5489 (~ 66%) protein annotated transcript hits (4451 with GO annotation, Fig. 4). Of those, 816 transcripts were differentially expressed with > 2 fold change (FC) and *p* < 10^− 10^ (389 in detox and 605 in cold, Table 2). Only transcripts with GO annotations and ORFs or interPro domains were considered. Differentially expressed transcripts had a mean OHR of 0.85 (Additional file 5: Figure S3B) and 65% of them were longer than the N50 value, indicating a higher degree of completeness than the entire transcriptome mean.

**Table 2:** Summary statistics of the DeepSuperSAGE analysis of 26 bp RNA tags after TrueQuant quality filtering (GenXPro)

	Total	In detox libraries^a^		In cooling libraries^a^	
		T	UT	C	UC
tags sequenced	12,010,268	1,331,970	2,815,748	3,378,922	4,483,628
UniTags^b^	87,509	30,438	45,922	59,479	64,846
transcripts best BLASTN hit for tags^c^	5489	5062		5278	
differentially expressed transcripts^c^	816	389		605	

^a^*T* TPA treated, *UT* untreated, *C* cooled, *UC* uncooled

^b^No. of unique tag sequences and the number that occurs in each library

^c^transcripts that where a best BLASTN hit (bit score > 40) for DeepSuperSAGE tags

Of the 816 differentially expressed transcripts, an overlap of 178 transcripts (~ 22%) was shared by both detox and cold experiments (Fig. 4). This may indicate a common evolutionary origin in trait regulation. Artificial induction of this overlap by the short freezing period of detox larvae (see methods) is unlikely because this was experienced by both detox treatment and control groups. Moreover, many of the common transcripts are regulated in opposite directions (Additional file 7: Table S3 and Additional file 8: Table S4). For example, regulation of metabolic pathways seems to be targeted in both traits, but by up- versus down regulation for detox and cold, respectively (Fig. 3). Moreover, ~ 83% of 4673 non-differentially expressed transcripts were trait overlapping, presumably referring to unspecific housekeeping and other not trait relevant genes. In contrast, the ~ 78% non-overlapping differentially expressed transcripts confirm our assumption of highly trait specifically regulated adaptive molecular mechanisms (Fig. 4).

The protein sequence descriptions of all BLAST transcript hits assigned to differentially expressed tags are listed in Additional file 7: Table S3 and Additional file 8: Table S4. Some differentially expressed unigenes had indifferent or oppositely regulated isoforms (Additional file 7: Table S3 and Additional file 8: Table S4), which indicates specific trait regulation by alternatively transcribed gene variants [18, 29], highlighting the relevance of functional characterization of paralogs and alternative protein variants. Enrichment analyses of functional GO, enzyme code (EC) and KEGG pathway annotations among up-regulated transcripts (Fisher’s exact tests, false discovery rate, FDR, correction cut-off = 0.05) are summarized in Table 3 (details for GO terms in Additional file 9: Table S5). Below, we discuss relevant candidate genes and enrichment results for TPA detoxification and cold hardiness in *H. euphorbiae*.

**Table 3:** Enriched GO terms among up-regulated transcripts (detox and cold)

Detox GO terms	REVIGO semantic category	Generic GO slim terms
Biological process		
0006629, 0016042, 0044255, 0044712, 0046486	lipid metabolism	lipid metabolic process
Molecular function		
0004175, 0004252, 0004806, 0008236, 0016298, 0017171, 0052689	serine hydrolase activity	peptidase activity
Cold GO terms	REVIGO category	Generic GO slim terms
Biological process		
0030029	actin filament-based process	biological process
0007010, 0007528, 0050808	cytoskeleton organization	cytoskeleton organization
0007498, 0055002	mesoderm development	anatomical structure development
0006820, 0006839, 0034763, 0051656, 0015711, 0015748, 0015858, 0015931, 0071705, 1901264, 1902047	nitrogen compound transport	transport, transmembrane transport
0001508, 0009118, 0019220, 0033238, 0042391, 0045979, 0051174, 0060249	regulation of membrane potential	homeostatic process, small molecule metabolic process, cellular nitrogen compound
0006591, 0006593	ornithine metabolism	small molecule metabolic process, cellular amino acid metabolic process
0006979, 0007626, 0009612, 0034599	response to oxidative stress	response to stress
Molecular function		
0005337, 0008509, 0008514, 0015291, 0015297, 0015301, 0015605, 0015932, 1901505, 1901677	carbohydrate derivative transporter activity	transmembrane transporter activity
0004099	chitin deacetylase activity	hydrolase activity
0004687, 0061650	myosin light chain kinase activity	kinase activity
0008073	ornithine decarboxylase inhibitor activity	enzyme regulator activity
0016627, 0016628, 0032440	oxidoreductase activity, acting on the CH-CH group of donors	oxidoreductase activity
0005212, 0008307	structural constituent of muscle	structural molecule activity

GO terms (biological process and molecular function) are grouped according to REVIGO semantic categories [48] and generic GO slim terms

### TPA detoxification

The three enzyme classes that are focus of this paper, esterases, P450s, and GSTs, are supplemented by lipases (LIP), since EC and KEGG enrichment revealed the enzyme triacylglycerol lipase (EC:3.1.1.3; LIP) and glycerolipid metabolism to be significantly over proportionally annotated among up-regulated transcripts. The pancreatic enzyme triacylglycerol lipase acts only on an ester-water interface and catalyzes the chemical reaction (IUBMB RN:R01369) triacylglycerol + H_2_O ⇌ diacylglycerol + a carboxylate (or fatty acid, RN: R02250; www.genome.jp/kegg-bin/show_pathway?ec00561+3.1.1.3). Capable of hydrolyzing the ester bond in this reaction, the enzyme may also be able to hydrolyze the ester bond of the phorbol ester TPA (see below). However, a study on the effect of TPA on the fatty acid metabolism in human cell cultures demonstrated a dramatic increase in the amount of cellular triacylglycerols as the TPA concentration is increased during treatment [47]. TPA would thus not increase or evoke lipid hydrolyzation, but rather the opposite pathway, lipid synthesis. Although no experiments have been undertaken study this effect in *H. euphorbiae*, it is supported by the anecdotal observation that *H. euphorbiae* larvae that ingested TPA weigh more upon pupation than control larvae. The role of TPA in the fatty acid and lipid metabolism should be studied in further detail, a first step being the verification of the differential triacylglycerol lipase expression via qRT-PCR.

A list of enriched GO terms within the 217 up-regulated detoxification transcripts (all transcripts in Additional file 7: Table S3) are summarized into REVIGO semantic categories [48] and generic GO slim categories in Table 3. The most up-regulated tag (tag48319; Additional file 7: Table S3; Log_2_FC 16.82) was annotated to the transcript mucin 4. This suggests that in addition to any enzymatic reactions, larvae could also decrease TPA uptake by the production of a physical layer of mucus. The most down-regulated tag (tag81144; Additional file 7: Table S3; Log_2_FC 17.90) was annotated to the transcript arylphorin alpha, a developmentally regulated larval storage protein suggested to serve as a store for amino acids for synthesis of adult proteins [49]. Arylphorins are hexamerins, large hemolymph-proteins mainly considered as storage proteins, but can also have other functions (e.g., cuticle formation, transport and immune response [50]). A possible explanation for the down-regulation upon oral TPA consumption could be that storage protein production is not necessary or even possible during the toxin-induced processes, since all amino acids are needed for proteins enabling the specific reaction. Overall, development, signaling, immune system (all biological processes), structural molecule activity und molecular regulation (all molecular functions) are suppressed (albeit slightly) upon TPA treatment (Fig. 3). It appears that the organism down-regulates non-essential processes during the acute up-regulation of detoxification-relevant processes. Larvae are observed to lie still in the few minutes after the first TPA ingestion.

Among biological processes, lipid metabolism was significantly over-proportionally annotated among up-regulated transcripts (Table 3). The corresponding up-regulated transcripts are annotated as lipases (*n* = 10; LIP), which mediate lipid digestion in insects, including Lepidoptera [51]. Lipases and carboxylesterases [18] (CESs: 3 transcripts up-regulated) play a role in TPA detoxification by fungi [52, 53], since the TPA structure has fatty acid moieties esterified to the hydroxyl group of the phorbol diterpene ground structure [54]. Among molecular functions, GO terms referring to serine hydrolase and peptidase activity were prominent and enriched among up-regulated transcripts. Hydrolases (*n* = 1) and peptidases/proteases (*n* = 10) contribute to general digestive activity, whereby serine proteases constitute about 95% of the digestive activity in lepidopterans and might reasonably be involved in processing toxic plant proteins [55]. However, TPA is not a protein, and we therefore focus the discussion on LIP, CES, P450 [56] and GSTs [57]. Five P450 and 2 GST transcripts were up-regulated and all other P450 and GSTs were downregulated (Additional file 7: Table S3). Only certain variants of these two large groups of enzymes appear to be involved in xenobiotic metabolism. Nevertheless, the majority (22 of 28) differentially expressed transcripts of these four ‘drug metabolism’ enzyme groups (CES, GST, LIP, P450) are up-regulated. In contrast, from all differentially expressed transcripts (detox experiment; Additional file 7: Table S3), only 69 of 175 ‘metabolism’ related transcripts are up-regulated, corroborating the importance of the four groups selected for detoxification in *H. euphorbiae*.

#### LIP

Lipases are esterases that hydrolyze long-chain acyl-triglycerides into di- and monoglycerides, glycerol, and free fatty acids at a water/lipid interface. The up-regulated triglyceride lipase H (Additional file 7: Table S3; Seq. ID C80796) corresponds to the (predicted) “lipase member H-A” from *B. mori* (GB Acc. No. XP_004932346; [58]). Similarly, the lipase referred to by the five transcripts C73752, s640, C54060, s638 and C45391 is specified as *B. mori* “pancreatic lipase-related protein 2” (PLRP2; XP_004929630.1). This enzyme participates in the cytotoxic activity of T-cells in mice [59]. By removing fatty acids, human PLRP2 process lipid antigens have a role in T cell immunity against mycobacteria [60]. Cellular TPA (see structure in [54]) uptake is largely based on partitioning of this lipophilic substance into the lipid phase of the cell membranes [61]. Thus, removal of the long chain fatty acid, presumably rendering the less lipophilic molecule phorbol 12-hydroxy 13-monoacetate (13-acetyl-phorbol, phorbol acetate [62]), would inhibit cellular uptake and could thus represent the first step of a detoxification pathway (see above). The transcript s961 refers to an “adipose triglyceride lipase” (AEJ33048.1) in *M. sexta*, whereby its insect homologue is called *brummer* [63]. This enzyme induces lipolysis in the *Bombyx* fat body during molting and pupation [63]. C78972 is referred to as “Abhydrolase” (ACB54944) from the peritrophic matrix from the larval *H. armigera* gut [64]. This term simply refers to an unspecified protein (in this case a lipase) containing an alpha/beta hydrolase fold, which is a catalytic domain found in a wide range of enzymes [65]. Lastly, s5060, corresponding to the “neutral lipase” in *H. armigera* (AFI64307.1), is a pancreatic lipase-like enzyme to which the above considerations also apply. In summary, we corroborate recording lipases as a group of enzymes possibly involved in TPA detoxification in *H. euphorbiae*.

#### P450

Cytochrome P450s are haem-thiolate proteins involved in the oxidative degradation of various compounds. They are particularly well known for their role in the degradation of environmental toxins and mutagens. P450 amino-acid sequences are extremely diverse, with levels of identity as low as 16%, but their structural fold has remained the same throughout evolution [66]. Of the cytochrome P450 superfamily, particularly the sub-clades CYP3, CYP4 and the mitochondrial clade are known for their role in detoxification processes in insects [18, 67, 68]. Among the 57 *H. euphorbiae* transcripts annotated as P450, 9 CYP3 and 4 CYP4 were found (with a mean length of 1290 bp). The remaining P450s belonged to other clades or were not assigned to any in the protein name (Additional file 7: Table S3). We discuss the five up-regulated variants. They were annotated with genes from the pest moths *Helicoverpa armigera*, *Operophtera brumata*, and *Spodoptera littoralis* [34, 56]. The transcript of a CYP324A1 (CYP3 clan) was most strongly up-regulated in *H. euphorbiae* (Additional file 7: Table S3; Log_2_FC 9.40). However, this gene was found only in tissues from the antennae of adult male moths [34]. This gene is thus not considered a prime detox candidate gene, but since it is up-regulated, its functional role in sphingid larvae deserves further examination. The up-regulated transcript with the sequence ID C83008 corresponding to CYP6AB31 was again described for an adult moth olfactory organ (*S. littoralis*; [56]), this gene is thus similarly not likely a prime detox candidate for *H. euphorbiae* larvae. The sequence ID C73934 corresponds to CYP9A21 in *B. mori* (GB Acc. No. BAM73828.1) which was found not to be expressed in the midgut, but rather the brain, epidermis, ovary and most interestingly, the fat body [69]. Some enzymes of the CYP9A group have been functionally thoroughly tested (especially CYP9A2) and their expression found to be induced by various xenobiotics in the *M. sexta* (Sphingidae) midgut (e.g., [70]). The sequence ID s5173 corresponds to CYP6BD6 from the *M. sexta* larval midgut (ADE05586.1; [36]). The CYP6 group of P450 enzymes are recently under focus again, since some variants are up-regulated in insects exposed to neonicotinoid insecticides [71]. Lastly, the sequence ID s5116 corresponding to CYP9A17 from *H. armigera* has been found to be overexpressed in the larval fat body, but not in the midgut [72]. Functional expression confirmed the protein to be involved in oxidative detoxification of pyrethroids [72], a major compound in household insecticides. In summary, we record CYP9A21, CYP6BD6 and CYP9A17 as candidate genes for further *H. euphorbiae* TPA detoxification analyses.

#### GST

GSTs are a group of multifunctional enzymes involved in xenobiotic and oxidative stress response [18, 73], which are also known to be inducible by environmental stress in *B. mori* [74]. In our dataset one (of two) up-regulated cytosolic GST (of 34 annotated) *H. euphorbiae* transcripts (glutathione S-transferase epsilon 2, GSTe2, length: 923 bp) belongs to the insect specific epsilon clade, which is expressed as gene families in *Anopheles gambiae* and *Drosophila melanogaster* and has also been found in *M. sexta* [57]. This is particularly relevant for detoxification (insecticide resistance) in insects [18, 57, 73], while the other up-regulated GST probably refers to some conserved physiological pathway [57] (sigma clade, encoded by a single gene found in a diverse range of species from nematodes to mammals). In the mosquito *A. gambiae* (Diptera), GSTe2 encodes an enzyme that has the highest levels of DDT dehydrochlorinase activity reported for any GST [75]. We thus record GSTe2 as a candidate gene for further *H. euphorbiae* TPA detoxification analyses.

#### CES

CESs have a variety of physiological functions in insects including dietary detoxification (CES clades A-C) [18, 38, 76, 77], to which 19 (2 highly expressed) of the 32 CES annotated *H. euphorbiae* transcripts (mean length of 19 detox relevant transcripts: 1358 bp) may refer. The two up-regulated CES (Seq. IDs s613 and s3415) were annotated with *B. mori* sequences. The first (GB Acc. No. AGG20205.1) was described as “expressed in silkworm midgut and induction responses by xenobiotics”, the second (GB Acc. No. XP_004925500.1) as “liver carboxylesterase-like”. Carboxylesterase activity on TPA would also hydrolyze the ester bond (IUBMB RN:R00630), possibly producing a metabolite with changed solubility properties (see above discussions of lipases). Both are thus detox relevant candidate genes.

### Cold hardiness

Seasonal cold hardiness refers to the capacity of an organism to survive cold winters by acclimation [22, 23], which is often achieved in insects by the accumulation of cryoprotectants and/or by metabolically depressed live stages (e.g., diapause), which is mostly linked to specific gene expression patterns [78, 79]. Differential gene expression of the cold treatment in *H. euphorbiae* is (to a large extent) marked by down-regulation (Fig. 4, Additional file 6: Figure S4A), with metabolic GO terms being most prominent (Fig. 3). Down-regulated transcripts include energy expensive respiratory proteins like NADH dehydrogenase, cytochrome oxidase and ATP synthase (Additional file 8: Table S4). GO terms enriched among down-regulated transcripts almost exclusively comprise metabolism, aerobic respiration and dissimilative functions (Additional file 9: Table S5). These results are strongly indicative of metabolic depression [29, 43]. As both our cold acclimated and uncooled control groups were diapause pupae, the metabolic changes appear to be triggered particularly by low temperature, instead of being determined by general developmental pathways. Our results are congruent with an earlier study showing strong metabolic rate reduction in overwintering compared to fresh *H. euphorbiae* diapause pupae [25], and highlights the importance of combined temperature and light regime patterns in triggering fully cold hardy life states in overwintering insects [20].

**Fig. 5:**
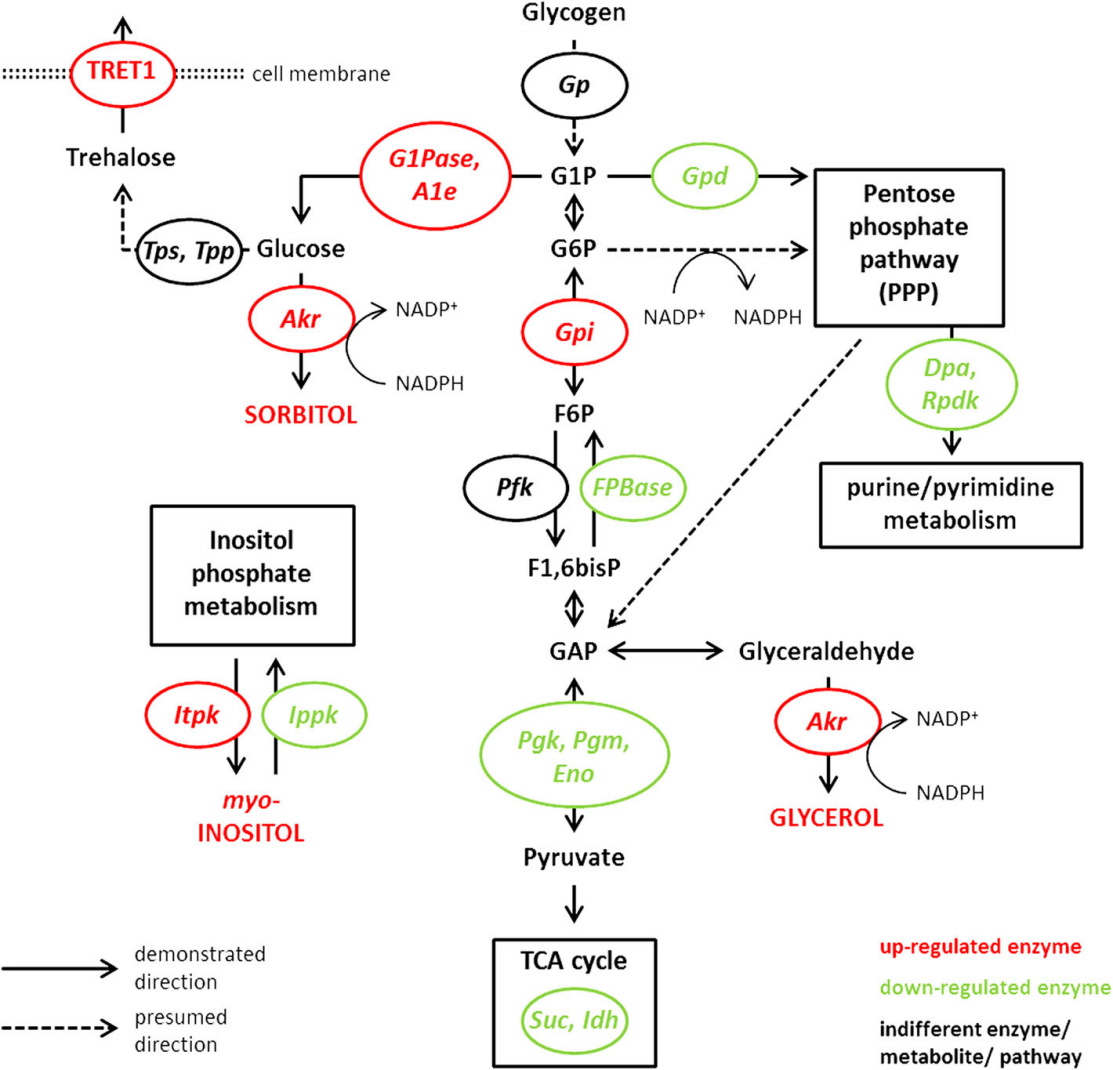
Simplified schematic diagram of inferred *H. euphorbiae* carbohydrate metabolism. Glycolysis runs down from glycogen degradation towards GAP and is then entirely directed into glycerol via *Akr*. Additionally, sorbitol may be produced from G1P originated glucose, though down-regulated *FPBase* most likely supports the glycerol pathway. The PPP is regulated in a way that most metabolites cycle back as GAP towards glycerol while providing NADPH reducing power. It is not known whether trehalose is produced from glucose at this phase of diapause, even though a TRET1 transcript was up-regulated. We presume that indifferently expressed transcripts are potentially regulated at the protein level via post-translationally signaling cascades as described in the text (details in [28, 29, 80]). Alternatively, regulation may have taken place in an earlier diapause phase or was too weak or tissue specific to be detected with our sampling and sequencing scheme. Protein legend: *A1e* – aldose-1-epimerase; *Akr* – aldo-keto reductase; *Dpa* – deoxyribose-phosphate aldolase; *Eno* – enolase; *FPBase* – fructose-1,6-bisphosphatase; *G1Pase* – glucose-1-phosphatase; *Gpd* – glucose dehydrogenase; *Gp* – glycogen phosphatase; *Gpi* – glucose-6-phosphate isomerase; *Ippk* – inositol polyphosphate phosphatase; *Idh* – isocitrate dehydrogenase; *Itpk* – inositol-trisphosphate 3-kinase; *Pfk* – 6-phospho-fructokinase; *Pgk* – phosphoglycerate kinase; *Pgm* – phosphoglycerate mutase; *Rpdk* – ribose-phosphate diphosphokinase; *Suc* – succinyl-CoA ligase; *Tpp* – trehalose-6-phosphatase; *Tps* – trehalose-6-phosphate synthase; TRET1 – trehalose transporter 1. Metabolite legend: G1P – glucose-1-phosphate; G6P – glucose-6-phosphate; F6P – fructose-6-phosphate; F1,6bisP – fructose-1,6-bisphosphate; GAP – glyceraldehyde-3-phosphate

Common cryoprotectants in insects are polyols and the insect blood sugar trehalose, mainly synthesized from glucose and glycogen via glycolysis and the pentose phosphate pathway (PPP) [24, 28, 29]. Gene expression shifts towards production of these cryoprotectants are indeed particularly marked in *H. euphorbiae* (Fig. 5; Additional file 8: Table S4), which was already indicated in early studies [4, 25, 26, 80]. The carbohydrate metabolism is thus our main focus here. Glycolysis enzymes *G1Pase*, *A1e*, *Gpi* and an *Akr* isoform are up-regulated (see enzyme and metabolite abbreviations in Fig. 5). The former three are involved in glucose production from the glycolysis metabolites G1P, G6P and F6P. *Gpi* also catalyzes glycolysis down-processing towards the GAP metabolite. *Akr* serves as polyol dehydrogenase and catalyzes polyol synthesis of both sorbitol from glucose and glycerol from GAP. In contrast, the gluconeogenetic enzyme *FBPase* was down-regulated, preventing a back-flow of GAP, which is a well-known regulatory switch towards glycerol accumulation in cold hardy insects [28, 78], and was, e.g., also down-regulated during diapause of the cotton bollworm *Helicoverpa armigera* [81]. Likewise the glycolysis enzymes *Pgk*, *Pgm* and *Eno* are down-regulated, preventing further processing of GAP into aerobic energy provisioning in the TCA-cycle (Fig. 5). Other down-regulated enzymes belong to the TCA-cycle itself (*Idh* and *Suc*), and to the PPP (*Gpd*, *Dpa* and *Rpdk*). *Gpd* competes with the polyol/sugar synthesizing pathways for glucose substrate, and *Dpa* and *Rpdk* process PPP products further into the purine/pyrimidine metabolism. Down-regulation results in back-circulation of the PPP products into glycolysis as GAP, providing substrate and NADPH reducing power for glycerol production [28, 80]. Additionally, *Itpk* synthesizing another polyol, *myo*-inositol was up-regulated in the inositol metabolism, whereas the degrading enzyme *Ipp* was down-regulated (Fig. 5).

Thus, glycerol appears to be the predominant polyol cryoprotectant in *H. euphorbiae* diapause pupae, produced from GAP via *Akr*. This has also been shown to be triggered by low temperature in the freeze-avoiding gall moth, *Epiblema scudderiana* [82], and by cryoprotective dehydration in the Antarctic midge, *Belgica antarctica* [83]. Glycerol is a potent osmolyte that, often in combination with dehydration, lowers the body liquid supercooling point (SCP) [24], and is most commonly found in cold hardy insects [29, 80], particularly with a freeze-avoidance strategy [22]. In fact, the lethal temperature of *H. euphorbiae* diapause pupae is strongly reduced [3], indicating a lowered SCP and freeze-avoidance [22]. Other polyols such as sorbitol and *myo*-inositol may play an additional role, as also found in the goldenrod gall fly, *Eurosta solidaginis* [80], and some Lepidoptera [84, 85], respectively.

An indicated accumulation of glucose via *G1Pase*, *A1e* and *Gpi* goes conform with a strong increase of glucose concentration in overwintering *H. euphorbiae* diapause pupae found in a biochemical study already nearly seventy years ago [4]. It is unclear whether glucose acts as cryoprotective as such, or serves as fuel for the production of other carbohydrates [83]. Many cold hardy insects increasingly metabolize the insect blood sugar trehalose from glucose [28], which stabilizes bi-lipid layer membranes during cryoprotective dehydration, a strategy to avoid freezing by staying in vapor pressure equilibrium with surrounding ice [28, 79]. Trehalose accumulation has indeed been found in *H. euphorbiae* in an early study [5]. However, even though we found a trehalose transporter TRET1 transcript up-regulated in our study, no trehalose synthesizing enzymes (*Tpp* or *Tps*, Fig. 5) were differentially expressed [28, 79, 83, 86, 87]. It is also surprising that we did not find differential expression of *Gp*, a key enzyme in breaking down glycogen as fuel for glycolysis and polyol biosynthesis (Fig. 5). Glycogen break down is strongly linked with an increase of cryoprotective carbohydrates like glucose, trehalose and glycerol, in cold hardy insects, including *H. euphorbiae* [88]. Missing regulation of some key genes in cryoprotective synthesis in our study may have two important causes:

First, most dramatic changes in trehalose and polyol metabolic enzyme expression are often found during early preparation of insect diapause [20, 81, 86, 87], while our samples had already established diapause at the time of treatment. Likewise, Stuckas et al. [2] did not find differentially expressed carbohydrate metabolic enzymes in cold acclimated *H. euphorbiae* diapause pupae, and cold exposure alone did not trigger cryoprotective protein expression in *B. antarctica* [83]. Thus, expression patterns in our study may rather represent specifically cold triggered changes during acclimation. Besides TRET1, we also find other up-regulated osmolyte transporters like aquaporin and sugar solute carriers (Additional file 8: Table S4), indicating the maintenance of osmotic homeostasis. Moreover, several cold enriched GO terms refer to transport/homeostatic processes and carbohydrate transporter activity (Table 3). Osmolyte transport is often up-regulated during cold acclimation [89, 90], as for example is aquaporin, a well-known glycerol transporter [91]. This and the apparently cold specific regulation of several transcripts related to glycolysis and PPP metabolism (see above) substantially complements and corroborates earlier findings from proteomic profiling in *H. euphorbiae* [2].

Second, transcript regulation does not necessarily lead to protein regulation, which may result in discrepancies between transcriptomic and proteomic studies [44]. Often proteins are post-translationally regulated via enzyme kinetic changes, without changes in expression level [29, 44, 82], which was shown for *Gp* being activated by cold in a silk moth [92]. In our study signaling related GO terms were numerous among up-regulated transcripts (Fig. 3). Various transcripts refer to proteins of second messenger signaling cascades of post-translational regulation, including 14-3-3 protein zeta, G-proteins, exportin, cAMP responsive transcription factors and Ca^2+^ dependent protein kinases, in combination with down-regulated Ca^2+^ binding proteins (Additional file 8: Table S4). Regulation in Ca^2+^ signaling has been shown in cold acclimating *Drosophila* [89] and rapid cold hardening *E. solidaginis* [93]. Furthermore, cAMP and Ca^2+^ dependent protein kinases are often involved in regulating insect carbohydrate metabolism [28, 29]. We also found up-regulation of splicing factors, eukaryotic translation factors and ribosomal proteins (Additional file 8: Table S4) indicating generally enhanced gene expression activity and regulation at several levels beyond the transcriptome in *H. euphorbiae*. Regulation in signaling and transcriptional/translational activity has also been found in various other diapausing insects [2, 39, 78, 87, 94]. In *H. euphorbiae* changes in enzyme kinetics are also apparent by a shift in the amino acid pool as indicated by enriched GO term processes of nitrogen compound and ornithine metabolism (Table 3). This may explain early findings of accumulated uric acid and free amino acids [26, 27] as product of metabolic change [95].

Further cold specific GO term and gene expression data in *H. euphorbiae* refer to stress and antioxidant activity. Under prolonged periods of cold exposure insects are subject to enhanced oxidative stress, and a molecular response upon cold acclimation is common [29, 78, 89, 90], which has already been extensively discussed for *H. euphorbiae* [2]. Here we can particularly add the discovery of up-regulated heat shock proteins (Hsp90, Hsp70B and small Hsp20, Additional file 8: Table S4). These protein protective chaperones are also well-known adaptations to cold [78, 79] and have also been found up-regulated, e.g., in the moths *E. scudderiana* [29] and *Grapholita molesta* [96], as well as in many other insects [78]. Additional transcriptomic changes in *H. euphorbiae* refer to cold specific re-arrangements of the cytoskeleton as a typical cold acclimation reaction in insects [2, 81, 89, 90].

## Conclusion

We provide the first draft transcriptome for *H. euphorbiae*, the HEC and the hawkmoth subfamily Macroglossinae. The transcriptome comprised 16,082 unigenes, and we were able to qualify sets of differentially expressed candidate functional genes of TPA detoxification and seasonal cold acclimation – two important ecological traits in the HEC. Genes of the LIP, P450, CES and GST families were the most promising detoxification candidates [18]. For cold acclimation, we primarily focused on the carbohydrate metabolism as major source of cryoprotectives [24, 28, 29]. Twenty protein candidates involved in glucose, glycerol, *myo*-inositol and potentially sorbitol and trehalose synthesis were identified (Fig. 5).

Some overlap between differently expressed detoxification and cold hardiness transcripts (Fig. 4) may indicate a common evolutionary origin of both traits from general cell metabolism and stress response [18, 28, 29], while a majority (~ 78%) of highly trait specificly regulated transcripts indicates subsequent specialization. In cold hardiness, additional pathways, such as post-translational protein modification, cell signaling and cytoskeletal rearrangement are relevant. In contrast, detoxification adaptation seems to be highly concentrated on metabolic pathways, as we find strong down-regulation, for example, for signaling, immune system and developmental pathways. The latter can be largely ruled out as an influence, because we only compared groups of the same developmental stages.

We emphasize that the conclusions drawn here may serve as basic working hypotheses for future studies and the identified candidates as subject of further evaluation. Transcriptome completeness, coverage and protein annotation success should be improved in the future. Quantitative confirmation of differential expression of the candidates by qRT-PCR (e.g., [40, 77, 83]) and tissue-specific expression tests may improve the transcriptome. For example, detoxification is particularly regulated in the midgut [36], and some diapause genes are known to be expressed in the brain [81]. A more complete and tissue-specific transcriptome may provide better insight into gene expression in different parts of the body. Lastly, the presumed cryoprotectives should be confirmed on a metabolic level and physiologically associated with a lowered SCP [84, 85, 95], which makes a cold-hardy phenotype.

We predict that candidate biomarkers identified likely contribute to a deeper understanding of mechanistic traits and shed some light on the complex species delineation and ecological adaptation within the HEC [6, 8, 9]. For example, it would be interesting to compare gene expression of *H. euphorbiae* to its Southern European relative *H. tithymali* which is more susceptible to TPA and cold [1, 12, 21] and shows introgression with *H. euphorbiae* at the species boundaries [9, 11]. Similar studies have revealed profound differences between closely related Lepidoptera lineages under different environmental conditions [39, 40]. The reference transcriptome provided here will help to achieve these goals, thus enabling a better understanding of underlying molecular evolutionary processes. It will certainly facilitate more detailed investigations of ecologically functional traits and their relevant genes that might affect ecological species isolation under divergent environmental conditions in the HEC and across Lepidoptera.

## Methods

### Animal sampling, rearing and treatment

For both experiments (TPA detoxification and cold hardiness) a treatment and control group of three *H. euphorbiae* individuals each was taken from an F1 laboratory breeding strain of two specimens originally sampled in summer 2009 in Slovakia (48°56′12″N, 18°06′06″E) [2]. Experimental *H. euphorbiae* were reared at room temperature (RT) under short day conditions (13 h light, 11 h dark) that lead to diapause pupae [12, 19]. Small larvae (L1 – L4 instars) were fed twice a day with fresh *E. segetalis* or *E. myrsinites* leaves, and L5 instars (having completed the last molt before pupation) were kept on an artificial diet recipe [1].

For the TPA detoxification experiment, L5 instar larvae of the treatment group were fed with TPA-spiked (0.5 – 2 mg solved in dimethyl sulfoxid, DMSO) artificial diet one to three days after molting (weight of larvae: 2.8 – 3.3 g). Control larvae of the same age (weight: 2.2 – 3.5 g) received the artificial diet with DMSO only [1]. Shortly after TPA feeding (after 5, 10 and 27 min) experimental and control larvae were sacrificed by cooling them down in a − 20 °C freezer and dissecting them to avoid tissue contamination with gut content of plant material. For the cold hardiness experiment, L5 instar larvae that fed on artificial diet without additives were allowed to pupate and kept at RT for two months to ensure that they were in diapause. Afterwards, the treatment group was subject to gradual cooling from room temperature to − 2 °C over 36 days, to simulate a natural cold acclimation regime [2] well above − 10 °C, the temperature with 50% mortality [3]. After subjection to experimental conditions, treatment and control pupae were shock-frozen and ground in liquid nitrogen with a mortar (control pupae at the time when cooling of the treatment group started). Of each of the four groups (larval TPA and pupal cooling treatments, and respective controls), merged tissue of the three biological replicates were preserved in RNA-later at − 20 °C for subsequent RNA isolation. Additionally, adult moth tissue was preserved for RNA isolation in the same manner to include the adult life stage. A soft tissue mix was taken from the inside of the head, thorax and abdomen of three adult individuals sampled in 2011 at the same geographic location.

### Reference transcriptome sequencing

An *H. euphorbiae* reference transcriptome (1-KITE library ID: INSinlTAYRAAPEI-56) was generated covering background and housekeeping gene expression, as well as the diversity of transcripts sampled from a species’ different life stages and different experimental conditions with respect to the two traits (TPA detoxification and cold hardiness). Optimized amounts of tissue of all four larval/pupal groups (experimental and control) and adults (about 10 mg for larval tissue, 200 mg for pupae and 20 mg for adults) were pooled to contribute similar amounts of RNA, and sent as one sample to BGI-Shenzhen, China. All steps from RNA isolation to transcriptome assembly were carried out at the BGI, following a standardized procedure as given here in short (details in Misof et al. [31]).

A total RNA amount of 130 μg with an RNA integrity number (RIN) of 7.2 was extracted from the tissue mixture using the TRIzol reagent (Invitrogen). A combined cDNA library was prepared by transcribing the Dynabead isolated and cleaved mRNA (SuperScript™II Reverse Transcriptase, Invitrogen). After end-repair, single adenine addition and adapter ligation, double stranded cDNA was selected for an insert size of 250 bp and PCR amplified with paired-end information. Subsequently, the cDNA library was fragment size verified and shotgun sequenced for 150 bp paired-end reads on an Illumina HiSeq2000 at BGI [31].

### Transcriptome assembly

Prior to the de novo assembly, low quality reads were removed from the raw data output if they had adapter contaminations, more than 10 ambiguous bases (Ns) or more than 50 low quality bases [31]. Transcriptome assembly from the remaining reads followed the de Bruijn graph algorithm as detailed in Misof et al. [31] by using the software SOAPdenovo-Trans-31 kmer version 1.01 [97]. Furthermore, the assembly was cleaned from remaining linker/adapter and foreign contaminations, such as vector, phage or bacterial sequences by checking against the UniVec database 7.1. All sequences > 200 bp that were included in the assembly of the final transcriptome were uploaded to the NCBI Transcriptome Shotgun Assembly (TSA) database and used for further analyses (deposited at DDBJ/EMBL/ENA/GenBank under the accession GCDK00000000; the version described in this paper is the first version, GCDK01000000; BioProject: PRJNA267878; BioSample: SAMN03247533).

The transcriptome assembly consisted of singleton contigs formed from linear k-mers and scaffolds linearized from paired-end connected contigs > 100 bp, together representing the total of sequenced transcripts. Moreover, if the paired-end data ambiguously connected contigs to two or more possible scaffolds, those were grouped to clusters of isoforms or alternative splice variants of transcripts [97], called isogroups. All of these, as well as transcripts that did not cluster, represent unigenes expressed in our *H. euphorbiae* samples.

### Transcriptome completeness

We used CLC Genomics Workbench version 6.5.1 (www.qiagenbioinformatics.com/products/clc-genomics-workbench/) to predict open reading frames (ORF) with a cut-off length of 60 codons as a proxy of the proportion of protein-coding regions in the assembled *H. euphorbiae* transcriptome. We further used CEGMA [45] to evaluate the transcriptome completeness by measuring the covered proportion of a set of 458 core eukaryotic genes that are considered to be expressed in a wide range of eukaryotes [45].

Lastly, we determined the Ortholog Hit Ratio (OHR) [42] as a measure of de novo transcriptome assembly completeness with respect to ortholog protein-coding regions in the related Lepidoptera model *B. mori*. We modified the OHR in a similar way as Gschloessl et al. [39], by using a combined Lepidoptera protein database of 129,871 sequences for OHR determination. This consisted of 14,623 and 16,254 protein sequences, respectively, of *B. mori* and *Danaus plexippus* (ftp://ftp.ensemblgenomes.org/pub/metazoa/release-20/fasta/), 70,688 Lepidoptera proteins from ButterflyBase (ButterflyBase_pro.fsa.gz) and 903 Lepidoptera proteins from SwissProt (www.uniprot.org/). Additionally, we added 27,403 protein sequences of *M. sexta* from Manduca Base (translated from the official gene set OGS2_20140407_proteins.fa). All sequences (downloaded September 2016) were used as reference database in a BLASTX search with our assembly as query and the same settings as above. The OHR was then determined as the ratio of the aligned length (bp without gaps) of a transcript to the total length of its best BLASTX hit, so that an OHR < 1 or > 1 indicates a shorter or longer transcript, respectively, compared to the ortholog coding region.

### Functional transcriptome annotation

All assembled transcripts were identified by retrieving best hits from a BLASTX search against the NCBI nr protein database (version 09/2016) using the Blast2GO software [98] with an *e*-value cut-off of *e* = 10^− 5^ and a sequence alignment similarity > 55%. Blast2GO was then used to functionally annotate all BLASTX hit transcripts with GO terms, GO slim terms, enzyme codes (EC) and metabolic pathways of the Kyoto Encyclopedia of Genes and Genomes (KEGG). The GO annotation was further optimized by merging annotations of a previously run InterProScan of protein domains (ANNEX augmentation) as implemented in Blast2GO.

### Differential gene expression and enrichment analysis

In order to detect differentially expressed transcripts in response to TPA and cold experiments, separate RNA samples were isolated (PerfectPure RNA tissue kit, 5Prime) from each of the four tissue mixtures (three replicates per mixture) of larval TPA and pupal cooling treatment and control groups, prior to pooling them for transcriptome sequencing. These four samples were sent to GenXPro (Frankfurt, Germany) for DeepSuperSAGE analysis [32] of short 26 bp mRNA tags for treatment and control of both experiments, generated via Illumina sequencing and filtered for artefacts (TrueQuant software, GenXPro), as described in Stuckas et al. [2]. This resulted in the four tag libraries: TPA treatment (T), untreated control (UT), cooled (C) and uncooled control (UC) (Table 2, Additional file 10: Table S6). In contrast to Stuckas et al. [2], the assembled transcriptome allows us a much more specific and efficient evaluation of differentially expressed *H. euphorbiae* transcripts, by performing BLASTN searches of all unique tag sequences (UniTags, Additional file 10: Table S6) per experiment (T/UT and C/UC) against the transcriptome (CLC Genomics Workbench). Most transcripts were best hits for several UniTags, partly with inverse expression directions. Thus, best hit transcripts were assigned only to the single UniTag with the highest alignment bit score and lowest *e*-value. Only hits with bit scores > 40 of an un-gapped alignment were considered.

Differential expression of transcripts was obtained from their assigned tags as Log_2_ of the tag’s fold change (FC), i.e., the ratio of normalized tag counts (tags per million, tpm) in the treatment and control libraries (zero counts were set as 0.05 tpm). A *p*-value was calculated from a probability distribution as given in eq. 2 of Audic & Claverie [99] (where N_1_ and N_2_ are the total numbers of tags in the libraries to compare, and *x* and *y* are the counts of a given tag in each library, as implememted in the GenXPro software, see also [2, 32]). To focus on the biologically most meaningful gene expression changes, and to avoid false positive discovery, we considered only transcripts with tags with an at least two-fold tpm difference (Log_2_FC < − 1 or > 1) and a stringent *p* < 10^− 10^ as significantly down- or up-regulated.

To identify differentially expressed gene functions, a GO term enrichment was run in Blast2GO for the up- and down-regulated transcripts of the TPA and cold treatments. Likewise, enzyme codes and KEGG pathways were enriched. For enrichments, we used Fisher’s exact tests with a Benjamini-Hochberg false discovery rate corrected *p*-value cut-off of 0.05. The GO term enrichment was further summarized into GO slim terms and REVIGO semantic categories [48].

## Additional files

Additional file 1: **Figure S1.** Percentage and number (in bars) of annotated transcripts. This is of a total of 18,080 transcripts with a cut-off of *e* = 10^− 5^ and 55% sequence similarity. BLASTX hits include GO term annotated hits. Those include KEGG hits, which in turn consist of transcripts annotated with enzyme codes and respective metabolic pathways. (JPEG 392 kb)

Additional file 2: **Table S1.** Table of 8289 *H. euphorbiae* transcripts with BLASTX hits (NCBI nr protein database version 09/2016). (XLSX 1824 kb)

Additional file 3: **Figure S2.** Distribution of species names among BLAST hits. The 20 most frequent species names affiliated with best BLASTX hits against nr are shown, making up (**a**) 8626 transcripts among the entire annotated *H. euphorbiae* assembly, and (**b**) 8219 (95%) among Lepidoptera matches only. (ZIP 259 kb)

Additional file 4: **Table S2.** List of 125 KEGG pathways and 513 different enzyme codes. Enzyme commission numbers (EC) and KEGG pathways are given; to which 2806 BLAST annotated *H. euphorbiae* transcripts have been mapped. (XLSX 53 kb)

Additional file 5: **Figure S3.** Distribution among ortholog hit ratio (OHR) classes. This is taken from BLASTX against Lepidoptera databases for (**a**) all protein annotated *H. euphorbiae* transcripts, and (**b**) only transcripts that were best BLASTN hits for differentially expressed DeepSuperSAGE tags in detoxification or cold treatments. (ZIP 33 kb)

Additional file 6: **Figure S4.** Distribution of DeepSuperSAGE tag frequencies and *p*-value vs. fold change (FC) of tags. Left panel: Normalized tag counts per million tags (tpm) in treatment and control libraries. Right panel: Relationship between tag FC (ratio of tpm counts) and *p*-value of significance. Up- and down-regulated tags (*p* < 10^− 10^, − 1 > Log_2_FC > 1) are marked in red and green, respectively. Of those, the best BLASTN hits to a transcript, according to bit score and e-value, were used to assess the transcript expression value. (**a**) cooled (C) vs. uncooled (UC) library. (**b**) TPA treated (T) vs. untreated (UT) library. (ZIP 946 kb)

Additional file 7: **Table S3.** Table of 389 detoxification related differentially expressed *H. euphorbiae* tags. Assignment with BLASTN transcript hits and annotated protein sequence descriptions (nr protein database version 09/2016). (XLSX 152 kb)

Additional file 8: **Table S4.** Table of 605 cold related differentially expressed *H. euphorbiae* tags. Assignment with BLASTN transcript hits and annotated protein sequence descriptions (nr protein database version 09/2016). (XLSX 169 kb)

Additional file 9: **Table S5.** Details of enriched GO terms among differentially expressed transcripts. GO terms (biological process and molecular function) are grouped according to REVIGO semantic categories and generic GO slim terms. (DOC 145 kb)

Additional file 10: **Table S6.** Total DeepSuperSAGE tag count. A total of 87,509 unique mRNA tags (UniTags) and their counts in the T, UT, C and UC libraries. (TXT 4497 kb)

## Abbreviations

°C: Degrees celcius
1KITE: 1000 Insect Transcriptome Evolution
A1e: Aldose-1-epimerase
Akr: Aldo-keto reductase
ATP: Adenosine triphosphate
BGI: Beijing Genomics Institute
bp: Base pairs
Ca^2+^: Ionized calcium
cAMP: Cyclic adenosine monophosphate
cDNA: Complementary deoxyribonucleic acid
CEGMA: Core Eukaryotic Genes Mapping Approach
CES: Carboxylesterase
CYP: Cytochrome P450
DDBJ: DNA DataBank of Japan
DDT: Dichlorodiphenyltrichloroethane
DeepSuperSAGE: Deep super serial analysis of gene expression
DNA: Deoxyribonucleic acid
Dpa: Deoxyribose-phosphate aldolase
EC: Enzyme commission number
EMBL: European Molecular Biology Laboratory
ENA: European Nucleotide Archive
Eno: Enolase
F6P: Fructose-6-phosphate
FC: Fold change
FPKM: Fragments per kilobase of transcript times millions mapped fragments
G1P: Glucose-1-phosphate
G1Pase: Glucose-1-phosphatase
G6P: Glucose-6-phosphate
GAP: Glyceraldehyde-3-phosphate
GO: Gene ontology
Gp: Glycogen phosphatase
Gpd: Glucose dehydrogenase
Gpi: Glucose-6-phosphate isomerase
GST: Glutathione S-transferases
GSTe2: Glutathione S-transferase epsilon 2
HEC: *Hyles euphorbiae* complex
Hsp: Heat shock protein
Idh: Isocitrate dehydrogenase
KEGG: Kyoto Encyclopedia of Genes and Genomes
L5: Fifth larval development stage
LIP: Triacylglycerol lipase
mRNA: Messenger ribonucleic acid
N50: Minimal length of the set of transcripts that makes up half of the total assembly length
NADH: Nicotinamide adenine dinucleotide
NCBI: National Center for Biotechnology Information
NGS: Next Generation Sequencing
OHR: Ortholog hit ratio
ORF: Open reading frame
PCR: Polymerase chain reaction
Pgk: Phosphoglycerate
Pgm: Phosphoglycerate mutase
PLRP2: Pancreatic lipase-related protein 2
PPP: Penthose phosphate pathway
Q20: Phred Quality Score
qRT-PCR: Quantitative reverse transcriptase polymerase chain reaction
REVIGO: Reduced visualized gene ontology
RIN: RNA integrity number
RNA: Ribonucleic acid
Rpdk: Ribose-phosphate diphosphokinase
SCP: Supercooling point
Suc: Succinyl-CoA ligase
TCA: Tricarboxylic acid cycle or Krebs cycle
TPA: Tetradecanoyl-phorbol-13-acetate
TRET1: Trehalose transporter 1
TSA: Transcriptome shotgun assembly
