# Supplementary figures and images for: Functional characterization of the Hyles euphorbiae hawkmoth transcriptome reveals strong expression of phorbol ester detoxification and seasonal cold hardiness genes
Source: Front Zool. 2018 May 1;15:20. doi: 10.1186/s12983-018-0252-2 (PMC5930835; doi:10.1186/s12983-018-0252-2)

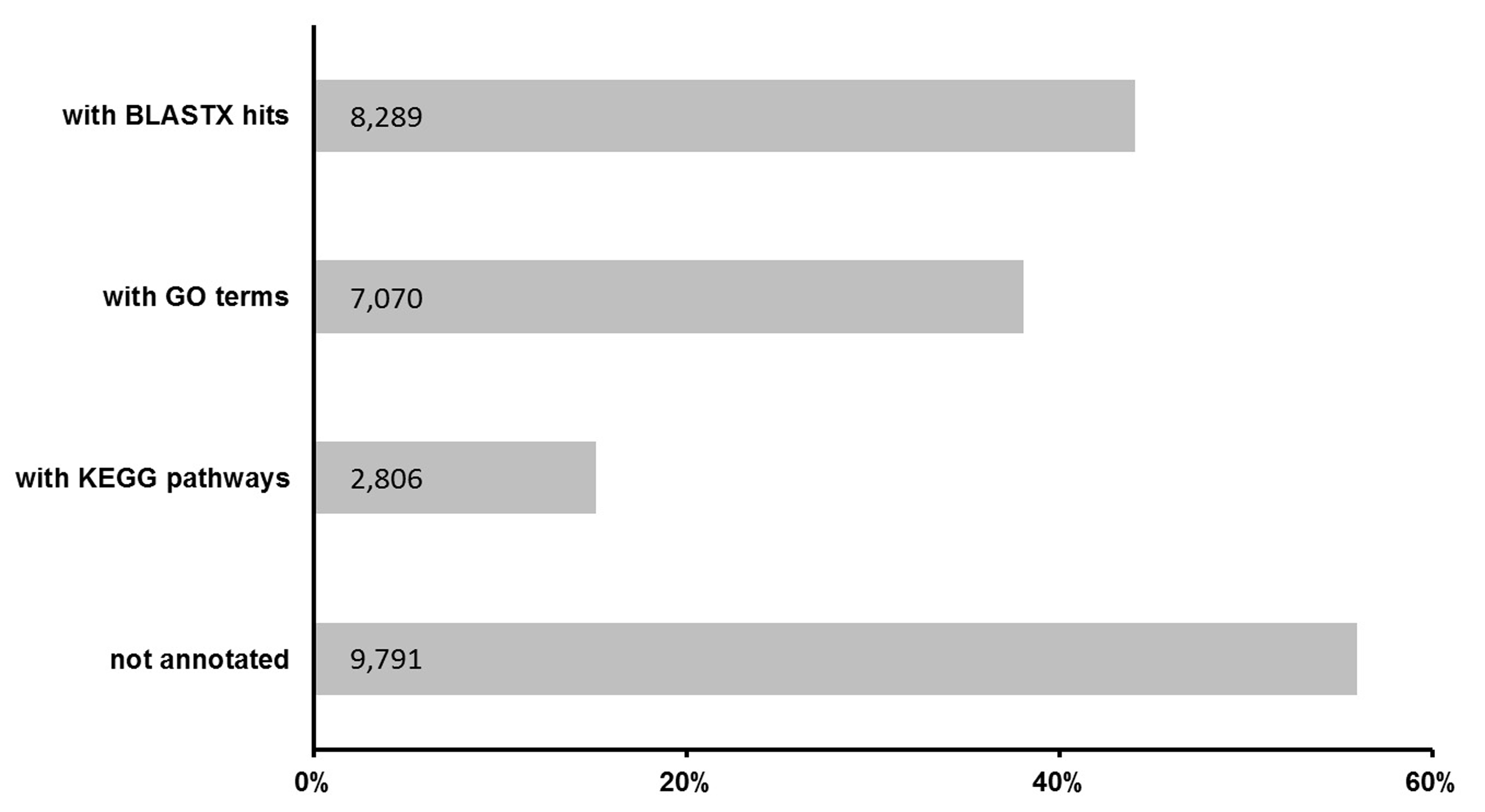

Supplement: Supplementary file 1 — Figure S1. Percentage and number (in bars) of annotated transcripts. This is of a total of 18,080 transcripts with a cut-off of e = 10− 5 and 55% sequence similarity. BLASTX hits include GO term annotated hits. Those include KEGG hits, which in turn consist of transcripts annotated with enzyme codes and respective metabolic pathways. (JPEG 392 kb) [file 12983_2018_252_MOESM1_ESM.jpg]

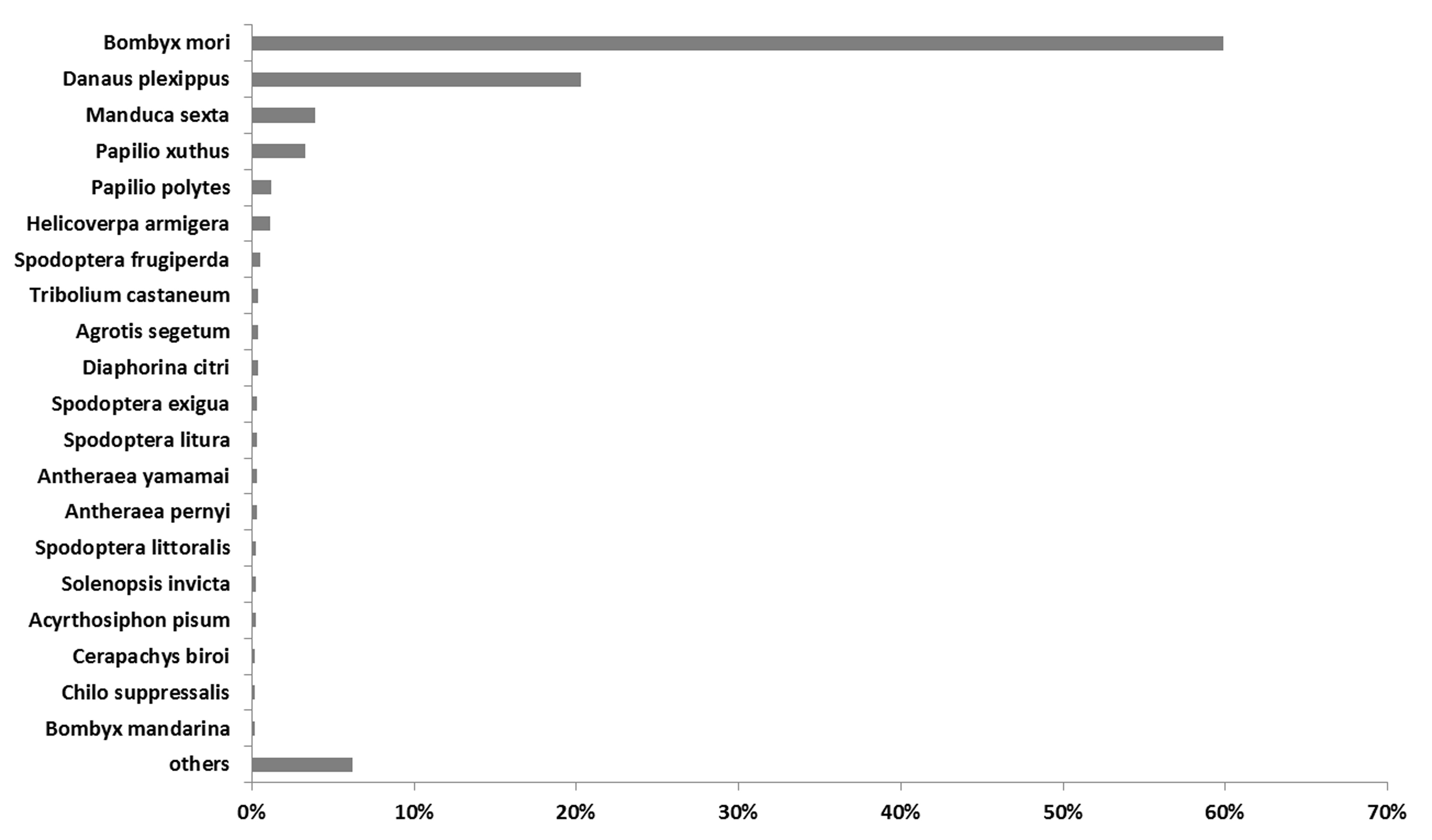

Supplement: Supplementary file 3 — Figure S2. Distribution of species names among BLAST hits. The 20 most frequent species names affiliated with best BLASTX hits against nr are shown, making up (a) 8626 transcripts among the entire annotated H. euphorbiae assembly, and (b) 8219 (95%) among Lepidoptera matches only. (ZIP 259 kb) [file 12983_2018_252_MOESM3_ESM.zip › Additional Fig S2a_Barth_etal.jpg]

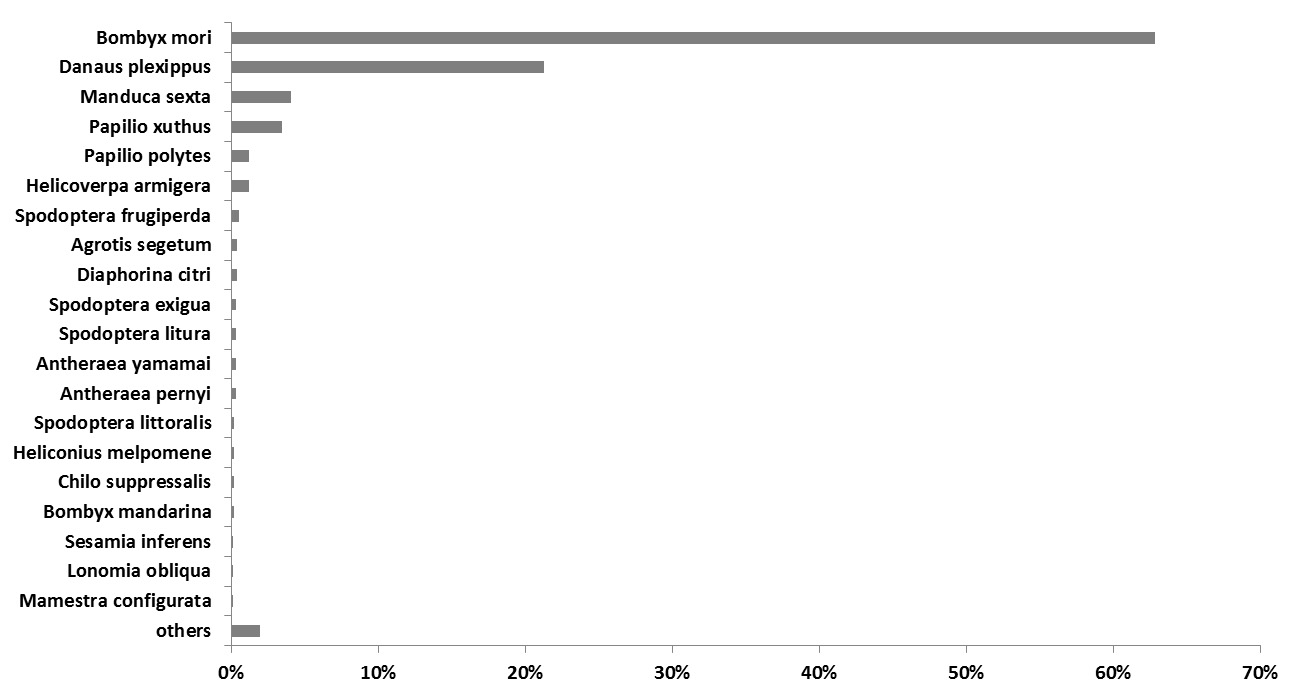

Supplement: Supplementary file 3 — Figure S2. Distribution of species names among BLAST hits. The 20 most frequent species names affiliated with best BLASTX hits against nr are shown, making up (a) 8626 transcripts among the entire annotated H. euphorbiae assembly, and (b) 8219 (95%) among Lepidoptera matches only. (ZIP 259 kb) [file 12983_2018_252_MOESM3_ESM.zip › Additional Fig S2b_Barth_etal.jpg]

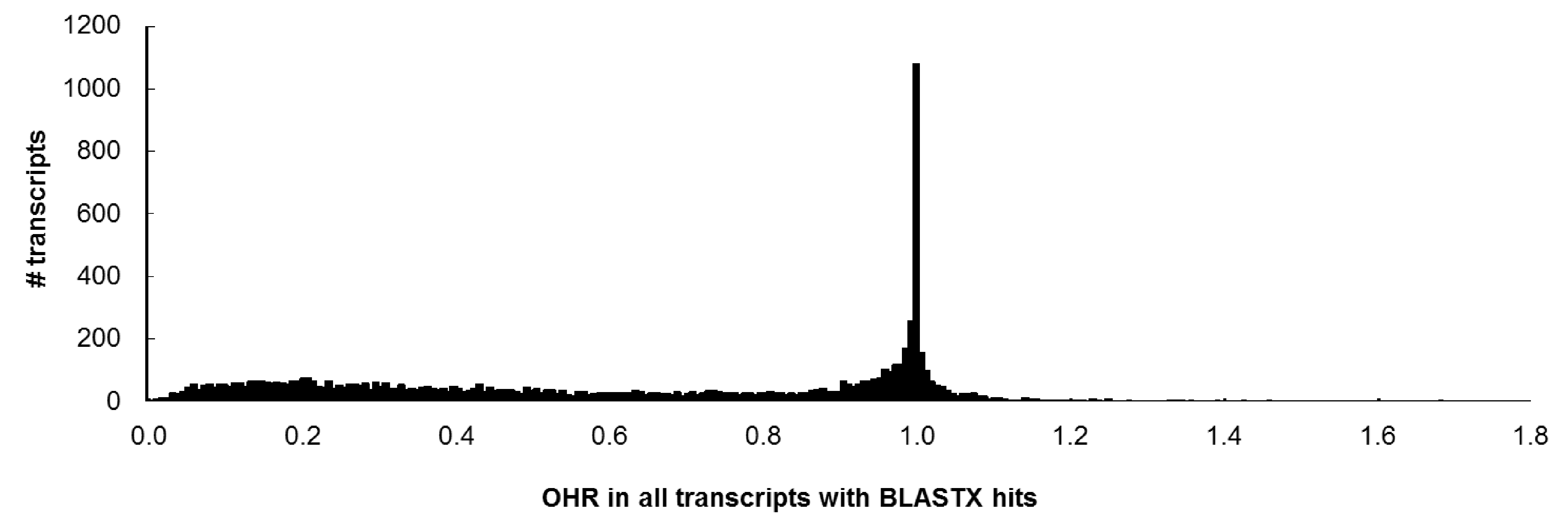

Supplement: Supplementary file 5 — Figure S3. Distribution among ortholog hit ratio (OHR) classes. This is taken from BLASTX against Lepidoptera databases for (a) all protein annotated H. euphorbiae transcripts, and (b) only transcripts that were best BLASTN hits for differentially expressed DeepSuperSAGE tags in detoxification or cold treatments. (ZIP 33 kb) [file 12983_2018_252_MOESM5_ESM.zip › Additional Fig S3a_Barth_etal.jpg]

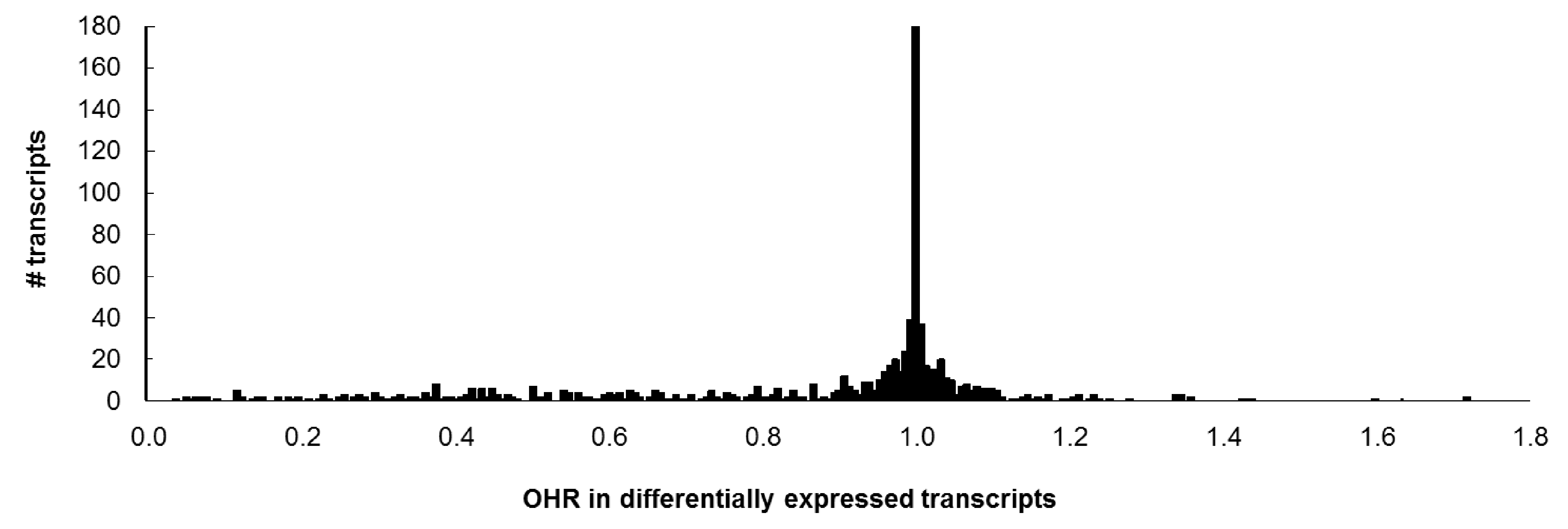

Supplement: Supplementary file 5 — Figure S3. Distribution among ortholog hit ratio (OHR) classes. This is taken from BLASTX against Lepidoptera databases for (a) all protein annotated H. euphorbiae transcripts, and (b) only transcripts that were best BLASTN hits for differentially expressed DeepSuperSAGE tags in detoxification or cold treatments. (ZIP 33 kb) [file 12983_2018_252_MOESM5_ESM.zip › Additional Fig S3b_Barth_etal.jpg]

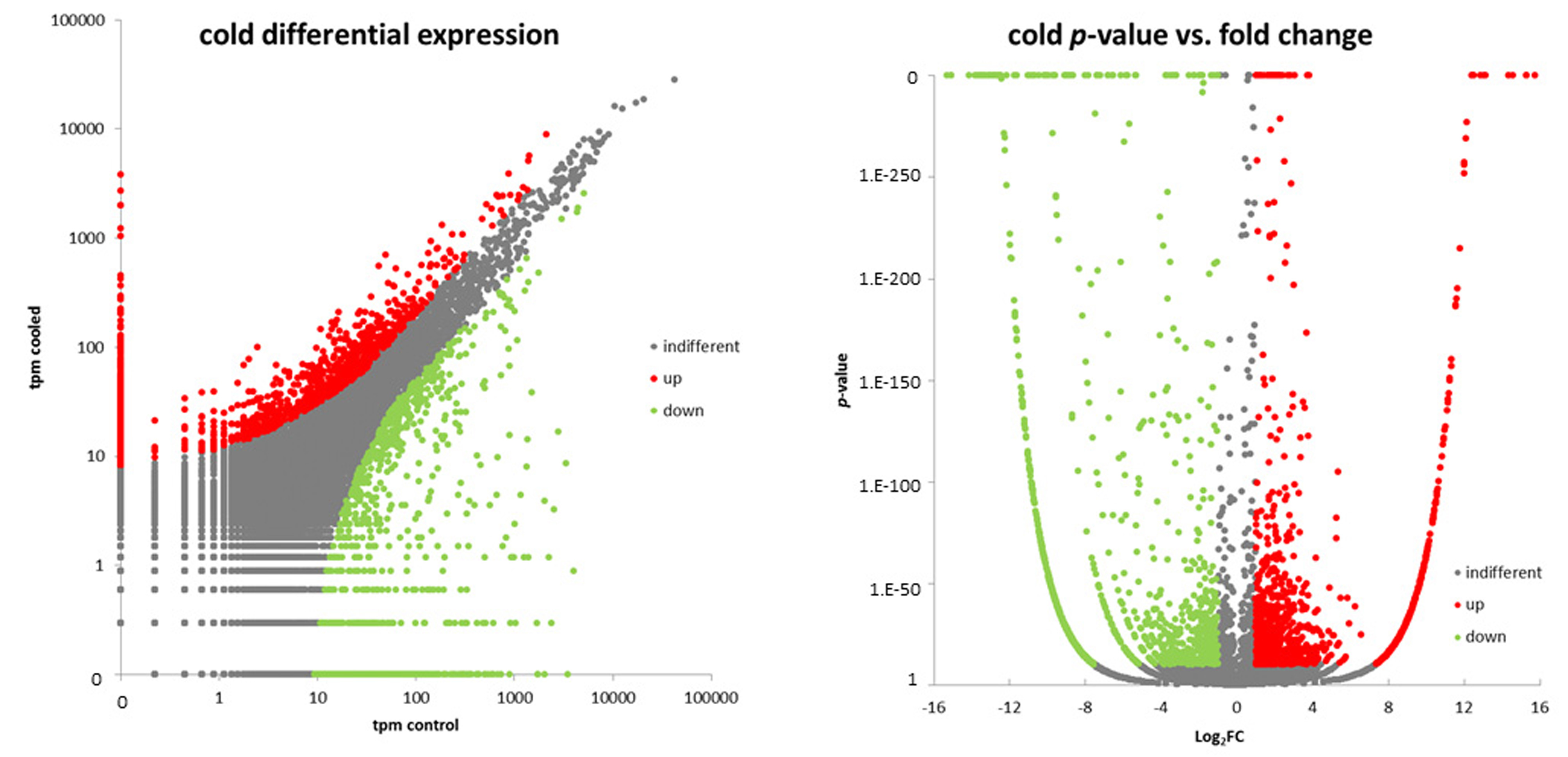

Supplement: Supplementary file 6 — Figure S4. Distribution of DeepSuperSAGE tag frequencies and p-value vs. fold change (FC) of tags. Left panel: Normalized tag counts per million tags (tpm) in treatment and control libraries. Right panel: Relationship between tag FC (ratio of tpm counts) and p-value of significance. Up- and down-regulated tags (p < 10− 10, − 1 > Log2FC > 1) are marked in red and green, respectively. Of those, the best BLASTN hits to a transcript, according to bit score and e-value, were used to assess the transcript expression value. (a) cooled (C) vs. uncooled (UC) library. (b) TPA treated (T) vs. untreated (UT) library. (ZIP 946 kb) [file 12983_2018_252_MOESM6_ESM.zip › Additional Fig S4a_Barth_etal.jpg]

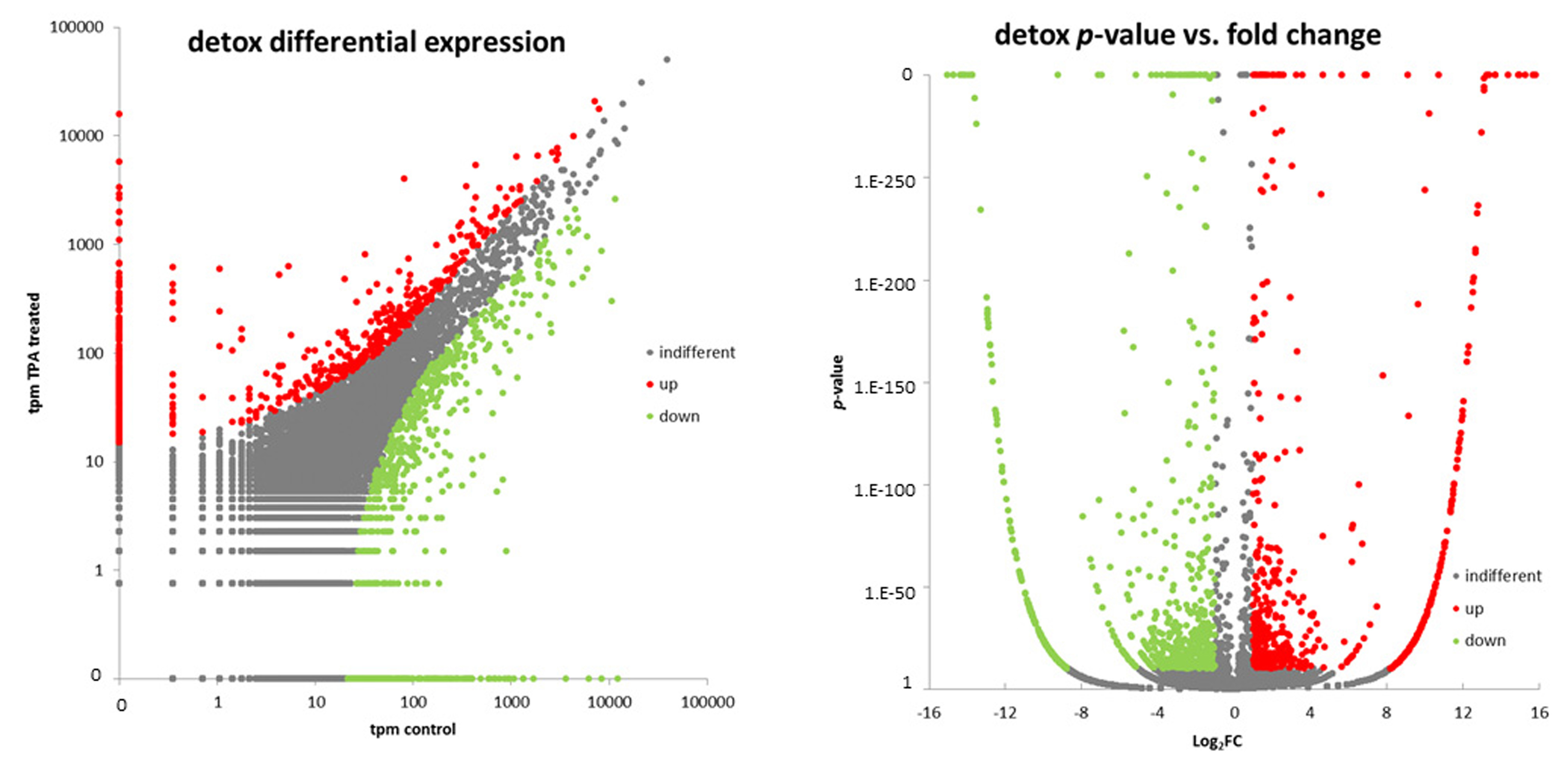

Supplement: Supplementary file 6 — Figure S4. Distribution of DeepSuperSAGE tag frequencies and p-value vs. fold change (FC) of tags. Left panel: Normalized tag counts per million tags (tpm) in treatment and control libraries. Right panel: Relationship between tag FC (ratio of tpm counts) and p-value of significance. Up- and down-regulated tags (p < 10− 10, − 1 > Log2FC > 1) are marked in red and green, respectively. Of those, the best BLASTN hits to a transcript, according to bit score and e-value, were used to assess the transcript expression value. (a) cooled (C) vs. uncooled (UC) library. (b) TPA treated (T) vs. untreated (UT) library. (ZIP 946 kb) [file 12983_2018_252_MOESM6_ESM.zip › Additional Fig S4b_Barth_etal.jpg]
