# Supplementary material for: Functional characterization of the Hyles euphorbiae hawkmoth transcriptome reveals strong expression of phorbol ester detoxification and seasonal cold hardiness genes
Source: Front Zool. 2018 May 1;15:20. doi: 10.1186/s12983-018-0252-2 (PMC5930835; doi:10.1186/s12983-018-0252-2)
Supplement: Supplementary file 9 — Table S5. Details of enriched GO terms among differentially expressed transcripts. GO terms (biological process and molecular function) are grouped according to REVIGO semantic categories and generic GO slim terms. (DOC 145 kb) [file 12983_2018_252_MOESM9_ESM.doc]

**Additional material**

**Functional characterization of the first *Hyles euphorbiae* hawkmoth *de novo* transcriptome assembly with focus on phorbol ester detoxification and cold hardiness**

M.B. Barth, K. Buchwalder, A.Y. Kawahara, X. Zhou, S. Liu, N. Krezdorn, A.K. Hundsdoerfer

**Additional Tables**

**Additional Table S5. Details of enriched GO terms among differentially expressed transcripts.** GO terms (biological process and molecular function) are grouped according to REVIGO semantic categories and generic GO slim terms.

| **Detoxification up-regulated GO terms** | | **REVIGO categories** | **Generic GO slim terms** |
| --- | --- | --- | --- |
| **Biological Process** | |  |  |
| 0044712 | single-organism catabolic process | lipid metabolism | catabolic process |
| 0016042 | lipid catabolic process |  | lipid metabolic process |
| 0006629 | lipid metabolic process |  |  |
| 0044255 | cellular lipid metabolic process |  |  |
| 0046486 | glycerolipid metabolic process |  |  |
| **Molecular Function** | |  |  |
| 0004806 | triglyceride lipase activity | serine hydrolase activity | molecular function |
| 0016298 | lipase activity |  |  |
| 0017171 | serine hydrolase activity |  |  |
| 0052689 | carboxylic ester hydrolase activity |  |  |
| 0004175 | endopeptidase activity |  | peptidase activity |
| 0004252 | serine-type endopeptidase activity |  |  |
| 0008236 | serine-type peptidase activity |  |  |
| **Detoxification down-regulated GO terms** | | **REVIGO categories** | **Generic GO slim terms** |
| **Biological Process** | |  |  |
| 1902850 | microtubule cytoskeleton organization involved in mitosis | microtubule cytoskeleton organization involved in mitosis | cell cycle, mitotic nuclear division, cytoskeleton organization |
| 1901215 | negative regulation of neuron death | negative regulation of neuron death | cell death |
| **Molecular Function** | |  |  |
| 0004553 | hydrolase activity, hydrolyzing O-glycosyl compounds | hydrolase activity, hydrolyzing O-glycosyl compounds | hydrolase activity, acting on glycosyl bonds |
| 0051060 | pullulanase activity |  |  |
| 0005212 | structural constituent of eye lens | structural constituent of eye lens | structural molecule activity |
| **Cold up-regulated GO terms** | | **REVIGO categories** | **Generic GO slim terms** |
| **Biological Process** | |  |  |
| 0030029 | actin filament-based process | actin filament-based process | biological process |
| 0007528 | neuromuscular junction development | cytoskeleton organization |  |
| 0050808 | synapse organization |  |  |
| 0007010 | cytoskeleton organization |  | cytoskeleton organization |
| 0007498 | mesoderm development | mesoderm development | anatomical structure development |
| 0055002 | striated muscle cell development |  |  |
| 0051656 | establishment of organelle localization | nitrogen compound transport | biological process |
| 0006820 | anion transport |  | transport |
| 0006839 | mitochondrial transport |  |  |
| 0015711 | organic anion transport |  |  |
| 0015748 | organophosphate ester transport |  |  |
| 0015846 | polyamine transport |  |  |
| 0015858 | nucleoside transport |  |  |
| 0015931 | nucleobase-containing compound transport |  |  |
| 0071705 | nitrogen compound transport |  |  |
| 1901264 | carbohydrate derivative transport |  |  |
| 0034763 | negative regulation of transmembrane transport |  | transport, transmembrane transport |
| 1902047 | polyamine transmembrane transport |  |  |
| 0001508 | action potential | regulation of membrane potential | biological process |
| 0019220 | regulation of phosphate metabolic process |  |  |
| 0042391 | regulation of membrane potential |  |  |
| 0051174 | regulation of phosphorus metabolic process |  |  |
| 0060249 | anatomical structure homeostasis |  | homeostatic process |
| 0009118 | regulation of nucleoside metabolic process |  | small molecule metabolic process, cellular nitrogen compound |
| 0033238 | regulation of cellular amine metabolic process |  |  |
| 0045979 | positive regulation of nucleoside metabolic process |  |  |
| 0006591 | ornithine metabolic process | ornithine metabolism | small molecule metabolic process, cellular amino acid metabolic process |
| 0006593 | ornithine catabolic process |  |  |
| 0007626 | locomotory behavior | response to oxidative stress | biological process |
| 0009612 | response to mechanical stimulus |  |  |
| 0006979 | response to oxidative stress |  | response to stress |
| 0034599 | cellular response to oxidative stress |  |  |
| **Molecular Function** | |  |  |
| 0005337 | nucleoside transmembrane transporter activity | carbohydrate derivative transporter activity | transmembrane transporter activity |
| 0008509 | anion transmembrane transporter activity |  |  |
| 0008514 | organic anion transmembrane transporter activity |  |  |
| 0015291 | secondary active transmembrane transporter activity |  |  |
| 0015297 | antiporter activity |  |  |
| 0015301 | anion:anion antiporter activity |  |  |
| 0015605 | organophosphate ester transmembrane transporter activity |  |  |
| 0015932 | nucleobase-containing compound transmembrane transporter activity |  |  |
| 1901505 | carbohydrate derivative transporter activity |  |  |
| 1901677 | phosphate transmembrane transporter activity |  |  |
| 0004099 | chitin deacetylase activity | chitin deacetylase activity | hydrolase activity |
| 0061650 | ubiquitin-like protein conjugating enzyme activity | myosin light chain kinase activity | molecular function |
| 0004687 | myosin light chain kinase activity |  | kinase activity |
| 0008073 | ornithine decarboxylase inhibitor activity | ornithine decarboxylase inhibitor activity | enzyme regulator activity |
| 0016627 | oxidoreductase activity, acting on the CH-CH group of donors | oxidoreductase activity, acting on the CH-CH group of donors | oxidoreductase activity |
| 0016628 | oxidoreductase activity, acting on the CH-CH group of donors, NAD or NADP as acceptor |  |  |
| 0032440 | 2-alkenal reductase [NAD(P)] activity |  |  |
| 0005212 | structural constituent of eye lens | structural constituent of muscle | structural molecule activity |
| 0008307 | structural constituent of muscle |  |  |
| **Cellular Component** | |  |  |
| 0005859 | muscle myosin complex | muscle myosin complex | cell, organelle, macromolecular complex |
| **Cold down-regulated GO terms** | | **REVIGO categories** | **Generic GO slim terms** |
| **Biological Process** | |  |  |
| 0055114 | oxidation-reduction process | aerobic electron transport chain | biological process |
| 0019646 | aerobic electron transport chain |  | generation of precursor metabolites and energy |
| 0016042 | lipid catabolic process |  | lipid metabolic process |
| 0006629 | lipid metabolic process |  |  |
| 0044255 | cellular lipid metabolic process |  |  |
| 0009820 | alkaloid metabolic process | alkaloid metabolism | biological process |
| 0009821 | alkaloid biosynthetic process |  | biosynthetic process |
| 0006022 | aminoglycan metabolic process | amino sugar metabolism | biological process |
| 0006040 | amino sugar metabolic process |  |  |
| 1901071 | glucosamine-containing compound metabolic process |  |  |
| 0005975 | carbohydrate metabolic process | carbohydrate metabolism | carbohydrate metabolic process |
| 0021952 | central nervous system projection neuron axonogenesis | central nervous system projection neuron axonogenesis | cell morphogenesis, cell differentiation, anatomical structure development |
| 0009593 | detection of chemical stimulus | detection of chemical stimulus | biological process |
| 0006508 | proteolysis | proteolysis |  |
| 0005976 | polysaccharide metabolic process |  | carbohydrate metabolic process |
| 0005984 | disaccharide metabolic process |  |  |
| 0006073 | cellular glucan metabolic process |  |  |
| 0009311 | oligosaccharide metabolic process |  |  |
| 0044042 | glucan metabolic process |  |  |
| 0044264 | cellular polysaccharide metabolic process |  |  |
| 0006012 | galactose metabolic process |  |  |
| 0018298 | protein-chromophore linkage |  |  |
| 0044710 | single-organism metabolic process | single-organism metabolism | biological process |
| **Molecular Function** | |  |  |
| 0000036 | ACP phosphopantetheine attachment site binding involved in fatty acid biosynthetic process | ACP phosphopantetheine attachment site binding involved in fatty acid biosynthetic process | molecular function |
| 0051192 | prosthetic group binding |  |  |
| 0003824 | catalytic activity | catalytic activity |  |
| 0008061 | chitin binding | chitin binding |  |
| 0020037 | heme binding | heme binding |  |
| 0016787 | hydrolase activity | hydrolase activity |  |
| 0031177 | phosphopantetheine binding | iron ion binding |  |
| 0005506 | iron ion binding |  | ion binding |
| 0005507 | copper ion binding |  |  |
| 0043169 | cation binding |  |  |
| 0046914 | transition metal ion binding |  |  |
| 0003997 | acyl-CoA oxidase activity | monooxygenase activity | oxidoreductase activity |
| 0004497 | monooxygenase activity |  |  |
| 0016634 | oxidoreductase activity, acting on the CH-CH group of donors, oxygen as acceptor |  |  |
| 0016705 | oxidoreductase activity, acting on paired donors, with incorporation or reduction of molecular oxygen |  |  |
| 0016491 | oxidoreductase activity | oxidoreductase activity |  |
| 0045735 | nutrient reservoir activity | nutrient reservoir activity | molecular function |
| 0004984 | olfactory receptor activity | olfactory receptor activity | signal transducer activity |
| 0004806 | triglyceride lipase activity | peptidase activity | molecular function |
| 0017171 | serine hydrolase activity |  |  |
| 0052689 | carboxylic ester hydrolase activity |  |  |
| 0004553 | hydrolase activity, hydrolyzing O-glycosyl compounds |  | hydrolase activity, acting on glycosyl bonds |
| 0004556 | alpha-amylase activity |  |  |
| 0008422 | beta-glucosidase activity |  |  |
| 0015926 | glucosidase activity |  |  |
| 0016160 | amylase activity |  |  |
| 0016798 | hydrolase activity, acting on glycosyl bonds |  |  |
| 0004175 | endopeptidase activity |  | peptidase activity |
| 0004177 | aminopeptidase activity |  |  |
| 0004252 | serine-type endopeptidase activity |  |  |
| 0008233 | peptidase activity |  |  |
| 0008236 | serine-type peptidase activity |  |  |
| 0008237 | metallopeptidase activity |  |  |
| 0008238 | exopeptidase activity |  |  |
| 0070011 | peptidase activity, acting on L-amino acid peptides |  |  |
| 0031409 | pigment binding | pigment binding | molecular function |
| 0046906 | tetrapyrrole binding | tetrapyrrole binding |  |
| 0004850 | uridine phosphorylase activity | uridine phosphorylase activity | transferase activity, transferring glycosyl groups |
| **Cellular Component** | |  |  |
| 0031224 | intrinsic component of membrane | anchored component of membrane | cellular component |
| 0031225 | anchored component of membrane |  |  |
| 0005576 | extracellular region | extracellular region | extracellular region |
| 0005615 | extracellular space | extracellular space |  |
| 0017177 | glucosidase II complex | glucosidase II complex | cell, cytoplasm, endoplasmic reticulum |
